# A multicentre, pragmatic, parallel group, randomised controlled trial to compare the clinical and cost-effectiveness of three physiotherapy-led exercise interventions for knee osteoarthritis in older adults: the BEEP trial protocol (ISRCTN: 93634563)

**DOI:** 10.1186/1471-2474-15-254

**Published:** 2014-07-27

**Authors:** Nadine E Foster, Emma L Healey, Melanie A Holden, Elaine Nicholls, David GT Whitehurst, Susan Jowett, Clare Jinks, Edward Roddy, Elaine M Hay

**Affiliations:** 1 Arthritis Research UK Primary Care Centre, Research Institute for Primary Care and Health Sciences, Keele University, Keele, Staffordshire ST5 5BG, UK; 2 Faculty of Health Sciences, Simon Fraser University, Burnaby, BC, Canada; 3 Centre for Clinical Epidemiology and Evaluation, Vancouver Coastal Health Research Institute, Vancouver, BC, Canada; 4 Health Economics Unit, Health and Population Sciences, University of Birmingham, Edgbaston, Birmingham B15 2TT, UK; 5 Staffordshire Rheumatology Centre, Haywood Hospital, High Lane, Burslem, Stoke-on-Trent ST6 7AG, UK

**Keywords:** Knee osteoarthritis, Knee pain, Physiotherapy, Exercise, Primary care, Adherence, Randomised controlled trial

## Abstract

**Background:**

Exercise is consistently recommended for older adults with knee pain related to osteoarthritis. However, the effects from exercise are typically small and short-term, likely linked to insufficient individualisation of the exercise programme and limited attention to supporting exercise adherence over time. The BEEP randomised trial aims to improve patients’ short and long-term outcomes from exercise. It will test the overall effectiveness and cost-effectiveness of two physiotherapy-led exercise interventions (Individually Tailored Exercise and Targeted Exercise Adherence) to improve the individual tailoring of, and adherence to exercise, compared with usual physiotherapy care.

**Methods/design:**

Based on the learning from a pilot study (ISRCTN 23294263), the BEEP trial is a multi-centre, pragmatic, parallel group, individually randomised controlled trial, with embedded longitudinal qualitative interviews. 500 adults in primary care, aged 45 years and over with knee pain will be randomised to 1 of 3 treatment groups delivered by fully trained physiotherapists in up to 6 NHS services. These are: Usual Physiotherapy Care (control group consisting of up to 4 treatment sessions of advice and exercise), Individually Tailored Exercise (an individualised, supervised and progressed lower-limb exercise programme) or Targeted Exercise Adherence (supporting patients to adhere to exercise and to engage in general physical activity over the longer-term). The primary outcomes are pain and function as measured by the Western Ontario and McMaster Osteoarthritis index. A comprehensive range of secondary outcomes are also included. Outcomes are measured at 3, 6 (primary outcome time-point), 9, 18 and 36 months. Data on adverse events will also be collected. Semi-structured, qualitative interviews with a subsample of 30 participants (10 from each treatment group) will be undertaken at two time-points (end of treatment and 12 to 18 months later) and analysed thematically.

**Discussion:**

This trial will contribute to the evidence base for management of older adults with knee pain attributable to osteoarthritis in primary care. The findings will have important implications for healthcare commissioners, general practitioners and physiotherapy service providers and it will inform future education of healthcare practitioners. It may also serve to delay or prevent some individuals from becoming surgical candidates.

**Trial registration:**

ISRCTN: ISRCTN93634563.

## Background

Knee pain in older adults is a common disabling problem, managed in the UK mostly in primary care [1]. Osteoarthritis (OA) is the most likely underlying diagnosis and refers to a clinical syndrome of joint pain accompanied by varying degrees of function limitation and reduced quality of life [2]. The most commonly affected peripheral joints are the knees and OA has been shown by radiography to be present in 70% of community dwelling adults aged 50 or more with knee pain [3]. Structural changes before radiography are common in the remainder [4]. Clinical trials and systematic reviews consistently show the benefit of exercise, in a variety of forms, for this patient group [5-9]. Exercise improves muscle dysfunction and reduces pain and disability without exacerbating joint damage [10]. It can reduce the risk of other chronic conditions [11] and improve the physical status of people with OA [12,13]. Physiotherapists are the largest group of exercise advisors for musculoskeletal problems in the UK National Health Service (NHS) and are therefore an appropriate group with which to develop and test strategies to improve outcomes from exercise with older adults with knee pain. Previous studies have shown that older adults with knee pain and physiotherapists involved in their treatment have concerns about the safety of exercise, do not consider exercise as an effective treatment for pain, do not focus on issues of exercise adherence and fail to translate traditional lower limb focused exercise into sustainable lifestyle changes [14].

The TOPIK trial [15] tested two primary care services for knee pain in older adults, an enhanced pharmacy review and community physiotherapy (based on advice and exercise) and compared these with advice and self-care alone. At 3 months, pain reduction was three times greater and functional improvement four times greater in those patients randomised to physiotherapy compared with advice alone, and more people obtained clinically meaningful changes [16,17] than those in the other two groups. Patients received, on average, four physiotherapy treatment sessions. There was evidence that the benefits in pain and function declined in the longer-term, suggesting that most patients require some form of monitoring or regular access to physiotherapy or exercise supervision for potential on-going benefit. The APEX trial [18,19], which incorporated a more intensive exercise intervention that was supervised and progressed over six treatment sessions, resulted in greater improvements in pain than the exercise programme in the TOPIK trial [15].

Similarly, other recent trials [20,21] and reviews [22] showed small to moderate, short-term reductions in knee pain and disability that are not sustained in the long-term. Exercise is clearly worth doing but we need to find out if, and how, the beneficial effects can be enhanced and maintained, crucial factors in the management of a chronic condition like knee osteoarthritis. Conversations with patients during telephone follow-ups in the APEX trial helped to explain why the effects of exercise might be sub-optimal [19]. Participants reported misconceptions about exercise in the presence of knee joint damage and pain, difficulty fitting the exercises into a daily routine, overly-complex exercise programmes, insufficient tailoring of the exercise programmes for their individual needs and, in some cases, they simply forgot to do the exercises.

Reviews highlight a lack of information about how to optimise exercise for this patient group [6-8]. Most studies are short-term, use limited measures of adherence and standardised exercise programmes [23]. Adherence, independent of exercise type, may be an important factor in the success of exercise interventions. There is evidence that better adherence to exercise improves pain relief [21] and disability [23] and that the addition of booster sessions may be helpful in maintaining positive effects on pain and function [22]. A UK consensus [24] identified adherence and tailoring of exercise to individuals as important research topics. In the general physical activity literature, a Cochrane review concluded that physical activity programmes that include patient goal setting, individually tailored and written exercise programmes, some professional guidance and ongoing support, may be the most effective approach [25]. In preparation for the BEEP trial, we conducted two studies. First, a Cochrane systematic review summarising the evidence to date about interventions to improve adherence with exercise in patients with chronic musculoskeletal pain [26]. Second, the Keele Attitudes and Beliefs Concerning Knee Pain (ABC knee) study investigated the exercise attitudes and behaviours of older adults with knee pain in the community (n = 611) and physiotherapists involved in managing older adults with knee pain (n = 538), to identify potential barriers to, and facilitators of, exercise for knee pain [14,27-29]. The findings of our Cochrane review, based on 42 trials with over 8,000 patients, suggested using a multifaceted programme combining educational and behavioural strategies to enhance exercise adherence, and incorporating individualisation of the exercise, follow-up and supervision to improve exercise adherence [26]. No one theoretical model underpinning exercise adherence was shown to be superior and the interventions that incorporated motivational strategies showed promise. A subsequent review by Bennell and Hinman [30] stated that the optimal exercise dosage is yet to be determined and an individualised approach to exercise prescription is required based on an assessment of impairments, patient preference, co-morbidities and accessibility. They suggested that maximising adherence is a key element dictating success of exercise therapy and that adherence can be enhanced by the use of supervised exercise sessions in the initial exercise period followed by home exercises. Bringing patients back for intermittent consultations with the exercise practitioner, or attendance at ‘refresher’ sessions was also suggested to assist long-term adherence and result in improved patient outcomes. Our ABC knee study showed that whilst physiotherapists use advice and exercise routinely for older adults with knee pain, they do not consider exercise to be an effective treatment for pain, have worries about its safety, provide care over relatively few treatment sessions thus reducing the capacity to adequately individualise, supervise and progress the exercise programme, and do not routinely follow-up patients to check adherence or to support the translation of lower-limb focused exercise into sustainable lifestyle changes in physical activity. Although patients are aware of risk factors (e.g. sedentary lifestyles), they find it hard to make and maintain appropriate lifestyle changes and express the desire for more support from health professionals [14,27-29]. Marks [31] describes the many factors that influence exercise adherence in patients with knee OA as either intrinsic (personal factors like self-efficacy, motivation, age, gender, disease status) or extrinsic (e.g. environmental, social factors or other lifestyle issues). These barriers can vary both over time and between individuals, meaning that one single approach to enhancing exercise adherence might not be as effective as an individually tailored approach. The World Health Organisation advocates an “adherence counseling toolkit” that can be used to systematically assess barriers and facilitators to adherence, and suggests that new interventions to enhance adherence are required [32].

Recent national and international clinical guidelines [2,24,33] support the overall effectiveness of exercise in knee OA, placing it as a key component of core treatment in primary care. However, there is a lack of evidence around the practical aspects of exercise delivery and maintenance, including how to support individuals to continue to exercise in the longer-term. UK guidelines from the National Institute of Health and Clinical Excellence (NICE) published in 2008 recommended future research should test ways to improve adherence with exercise [34] and the Chartered Society of Physiotherapy ranked testing ways to help patients incorporate exercise behaviours in their everyday life and increase the long-term effects of exercise as the top musculoskeletal research priority in its national priority setting exercise in 2010 [35].

The Benefits of Effective Exercise for knee Pain (BEEP) trial is a logical consequence of recent primary care trials, systematic reviews and guidelines for knee pain in older adults, which consistently support exercise-based interventions, but highlight the short-term, small to moderate-sized benefits [2,6]. The overall aim of the BEEP trial is to test, in older adults with knee pain attributable to OA, whether pain and function outcomes can be improved through changing the characteristics of the exercise programme in comparison to usual physiotherapy care. The research hypotheses are i) a physiotherapy-led individualised, supervised and progressed lower limb exercise programme is superior to usual physiotherapy care and ii) a physiotherapy-led intervention targeting exercise adherence in the longer-term and supporting the transition from lower limb exercise to general lifestyle physical activity is superior to usual physiotherapy care. Secondary objectives are to compare the cost-effectiveness of the two exercise interventions compared to usual care and investigate differences in knee pain-related perceptions and expectations, as well as exercise adherence and physical activity. A linked qualitative study exploring participants’ views of the interventions to inform interpretation of the trial results will also be conducted.

## Methods/design

### Design

This is a multi-centre, pragmatic, three-parallel group, assessor-blind, superiority, individually randomised controlled trial comparing two physiotherapy-led exercise-based interventions versus usual physiotherapy care, with embedded qualitative interviews with a subsample of participants. In preparation for this trial, we conducted a small pilot study in two NHS Primary Care Trusts (PCTs) to investigate the feasibility of training physiotherapists in the new treatment approaches, the acceptability of the treatments to patients and physiotherapists and to test the processes for the main trial.

For the main trial, participants are randomised (independently) at each treatment site with block randomisation at a 1:1:1 ratio to usual physiotherapy care (UC), individually tailored exercise (ITE) and targeted exercise adherence (TEA). 500 participants will be recruited over a period of 18 months. Each participant’s involvement with the trial is for 36 months, during which time they will all have access to usual primary care. The primary outcome is assessed 6 months from randomisation, but no analyses will be undertaken until the 18 month follow-up time-point.

### Setting

Participants are recruited from up to 100 general practices and their local physiotherapy services in the West Midlands and North West regions of the UK. Treatments are delivered within physiotherapy centres in up to 6 NHS Primary Care Trusts (PCTs) within the geographical regions of the Primary Care Research Network (PCRN) of the West Midlands North and the North West. The practices and treatment centres include a mix of urban/suburban/semi-rural/rural settings.

### Participants

Participants are eligible for inclusion if they are 45 years old and over, have current knee pain and/or stiffness in one or both knees, are able to read and write in English, are willing to participate, are able to give full informed written consent and have access to a telephone (for minimum data collection). We are deliberately choosing not to restrict the BEEP trial to people with radiographically diagnosed knee OA in order to reflect current clinical practice and national guidance [2] in which treatment choices are made on the basis of symptoms rather than on radiographic findings. We are thus including people typical of those seen in primary care. Patients recruited through the population survey and the record reviews at participating general practices will have a Chronic Pain Grade [36] severity of between 2 to 4, determined through a brief postal screening survey. This is to ensure that participants will have a mean level of pain and functional difficulty similar to those patients referred to physiotherapy (determined from our previous trials [15,19] and that the interventions will be suitable for such patients. Chronic Pain Grade 2 to 4 categories have also been reported as a clinically significant group of knee pain patients in other research studies [36-38].

Exclusion criteria are as follows: those with potentially serious pathology (such as inflammatory arthritis, malignancy), those who have had a total hip or knee replacement on the affected side, those who are on a waiting list for a total knee or hip replacement, those for whom their knee problem was caused by a recent trauma (sports injury, fall or accident), those for whom exercise interventions are contra-indicated (such as those with unstable cardiovascular disorders, severe hypertension, unstable angina or congestive heart failure), those who have received an exercise programme from a physiotherapist or a knee joint injection in the last three months, those residing in nursing home accommodation, those who are so severely physically restricted that they cannot get to the physiotherapy treatment centres and those who have a close family member already participating in the BEEP trial. Normal recreational involvement in physical activity will not be an exclusion criterion.

### Invitation, recruitment, consent and randomisation

#### Identification of potentially eligible participants

**Figure 1:**
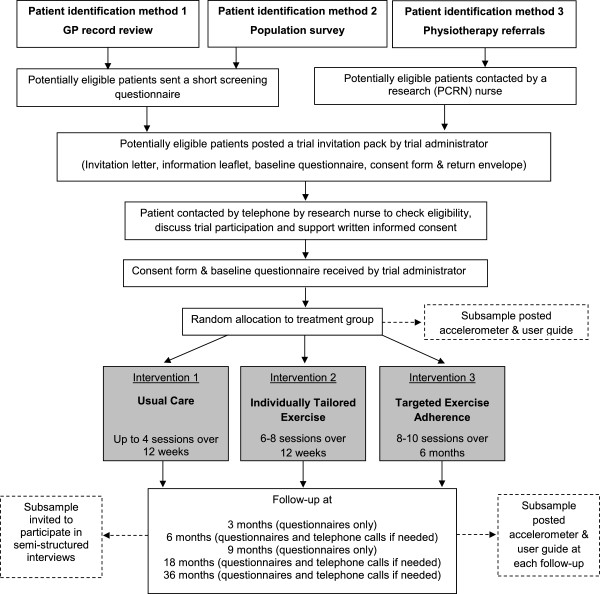
Flow diagram summarising participant recruitment in the BEEP trial.

The key learning from the pilot study which recruited participants only from those patients who were already referred by their General Practitioner (GP) to physiotherapy services was the need to increase recruitment to identify all potentially eligible participants. Therefore, in the main BEEP trial participants will be identified in one of three ways: (1) from general practice computer record reviews to identify those who have consulted for knee pain in the last 12 months, (2) from a population survey of older adults registered with participating practices and (3) from patients referred from their general practice to physiotherapy services for knee pain. These will proceed in parallel and are described in more detail below. Duplication checks will ensure that eligible participants are not invited to the BEEP trial more than once. See Figure 1 for a summary flowchart of the methods to identify and recruit participants.

(1) **General Practice record review:** Members of the Primary Care Research Network (PCRN) informatics team contracted to work in the participating general practices will screen computer records, for adults aged 45 years and over who have consulted with knee pain in the last 12 months at the practice. The electronic screen will identify patients based on the 14 most frequent knee pain related Read codes that have been informed by our previous research [39]. Read codes are the standard clinical terminology system that GPs enter onto their computer systems in the UK, identifying patients’ clinical symptoms and diagnosis. In addition, the electronic screening protocol will exclude those with potentially serious pathology (e.g. inflammatory arthritis such as rheumatoid arthritis, malignancy etc) and those in nursing home accommodation. GP’s will be invited to screen the sample list and exclude those patients whom they consider inappropriate to be invited to participate in the trial. PCRN staff will then administer the mailing of a short screening questionnaire to check eligibility and determine the patient’s Chronic Pain Grade classification [36]. Patients recruited through this method must have a Chronic Pain Grade severity of between 2 to 4. The final section of the brief screening questionnaire will ask patients whether they would be happy to give their consent for further contact. Patients will return their screening questionnaires to the Arthritis Research UK Primary Care Centre and only those who are eligible and consent to further contact will form the sample that is sent information about the BEEP trial.

(2) **Population survey:** Older adults (aged 45 years and over) registered with participating general practices will be mailed with a short screening questionnaire to identify potentially eligible trial participants. GP’s will be invited to screen the mailing list and exclude those patients whom they consider inappropriate to be invited to participate in the trial. This screen will be used to identify those in the community with knee pain of sufficient severity [36] to be included in the BEEP trial, and to exclude those who do not meet the eligibility criteria. Patients recruited through this method must have a Chronic Pain Grade severity of between 2 to 4. The final section of the brief screening questionnaire will ask older adults registered with the practice whether they would be happy to give their consent for further contact. Those who are eligible and consent to further contact will form the sample that is sent information about the BEEP trial.

(3) **Physiotherapy service referrals:** Older adults with knee pain referred by their GP or who self refer to participating physiotherapy services will be first screened, by a member of the physiotherapy service team, for key eligibility criteria (aged 45 years and over, with knee pain and/or stiffness). Those that are potentially eligible will be contacted by a PCRN research nurse to find out if they are willing to receive further information about the BEEP trial.

The three recruitment methods will proceed in parallel until the sample size required is reached, although methods 1 and 2 will be operationalised in different GP practices.

#### Recruitment and consent of participants to the BEEP trial

From identification methods (1), (2) and (3) above, those who meet the eligibility criteria and who agree to further contact will be posted information about the BEEP trial (a cover letter, a participant information sheet (PIS) (See Additional file 1 for a copy of the PIS), a baseline questionnaire, a consent form and a freepost return envelope). No less than 48 hours after receiving the posted information, a PCRN nurse will telephone the person to further check and confirm eligibility. This check will cover the full eligibility criteria in order to ensure that only those who meet these criteria are then recruited to the trial. In addition, the nurse will screen out individuals with known unstable cardiovascular disorders and those who have such severely restricted mobility that they would not be able to get to the physiotherapy clinics for treatment. All potentially eligible participants will have the opportunity to discuss the trial with the research nurse prior to deciding whether or not to participate. Those who wish to take part in the trial will be asked to sign and date the written consent form, supported by the research nurse over the telephone, and return it along with their completed baseline questionnaire to the nurse in a pre-paid envelope. Those who do not wish to take part will be asked to indicate this on the consent form and to return it to the nurse in the pre-paid envelope provided. This consent process was tested in the pilot study and regular audits of the nurse telephone calls will form part of the quality assurance procedures of the BEEP trial. It is possible, based on this consent method, that a very small number of participants in the BEEP trial will be found to be subsequently ineligible since it is only after consent that participants have a detailed physical assessment by a BEEP trial physiotherapist. Examples of this are expected to include a very small number of participants who have radiating leg pain from a spinal problem or a hip joint problem with referred pain in the area of the knee.

Participating general practices will be supported to assist with identification of potentially eligible participants for the BEEP trial through small practice payments to reimburse their time for screening patient lists and physiotherapy services will be supported to participate through financial reimbursement for the time taken out from service delivery for the training programme and additional time for BEEP treatments. Participants will not receive any financial incentives to return screening questionnaires, to take part in the trial or to return their follow-up questionnaires.

#### Randomisation and allocation concealment

Following receipt of a signed consent form and baseline questionnaire, the BEEP trial administrator will randomise the participant using a computer-generated randomisation schedule provided by the Musculoskeletal Clinical Trials Unit (CTU) at Keele University, password-protected to ensure that research nurses and trial statisticians remain blind to treatment allocation. Participants will be individually randomised to one of the three treatment groups using random permuted blocks of size 3. To ensure that patients at each physiotherapy clinic have a chance of receiving any of the interventions, randomisation will be stratified by physiotherapy clinic. Following randomisation, the trial co-ordinator will liaise with the appropriate physiotherapy clinic to arrange the first appointment for each trial participant. The patient will then be informed in writing of the date, time and location of their first appointment in the physiotherapy clinic. The GP of each trial participant will be sent a letter to confirm that their patient is taking part in the BEEP trial. Thus our procedures ensure baseline data are collected prior to randomisation, that the allocation is concealed until after the patient has been recruited into the trial and until the moment of randomisation and that the person assigning participants to intervention groups (study administrator) has no involvement in the eligibility screen, consent or treatment processes.

### Sources of bias

Selection bias at recruitment will be avoided by separating the processes of determining patient eligibility and treatment allocation and by using random permuted blocks overseen by the CTU, not allowing physiotherapists assessing and treating patients to predict the next allocation in their clinic. Trial participants will know they are having physiotherapy-led exercise and brief details about the three interventions (see Additional file 1 PIS). It is not possible to blind physiotherapists but they will deliver treatment to participants in only one of the three intervention groups. A PCRN nurse blind to treatment allocation will obtain informed consent and oversee the collection of baseline and follow-up questionnaire data, and will collect minimum data over the telephone where necessary. An evaluation of the success of nurse blinding procedures will be completed and a procedure for reporting incidents where blinding has been compromised will be in place.

Data entry, coding, security, storage and management will follow the standard operating procedures in the CTU at Keele University. Data enterers will receive training in line with CTU procedures and will be blind to the identification of the three intervention groups. Random 10% data entry accuracy checks will be conducted at regular intervals, data accuracy will be audited and accuracy rates recorded. The trial statistician (EN) will remain blind until after the creation of a locked analysis dataset at 18 months follow-up and the completion of the primary and secondary analyses.

Comparing available variables between consenting and non-consenting individuals, trial participant withdrawals and completers will be carried out to evaluate external validity. All participants will be free to withdraw from the trial at any time without having to give any explanation. Where possible, we will collect information about the reasons for withdrawal from the trial. Using validated outcome measures will reduce measurement error. Treatment will be recorded by physiotherapists in standardised formats and audits of these case report forms will be undertaken throughout. Feedback to physiotherapists delivering the interventions will be provided, where necessary, so they can modify and improve the delivery of the interventions. Each intervention is supported by a specific protocol and documentation, developed for the BEEP trial and previously tested in the pilot study.

### Interventions

The interventions will be delivered in participating physiotherapy centres in up to 6 NHS Primary Care Trusts (PCTs). All patients will receive an advice and information booklet (which will include information about the value of specific lower limb and general exercise and simple self-help messages such as the use of analgesics, and home heat therapy for pain relief, see Additional file 2 for a copy of the advice booklet) and a home exercise programme. Different physiotherapists delivered each of the three interventions. Each of the three intervention groups are detailed below with supporting explanation and justification (see Table 1 for a summary of the interventions). They are all examples of complex interventions as they involve a number of separate but interacting components that are likely to be important to the success of the intervention [40]. Whilst the key outcomes are knee pain and disability, the interventions are all behaviour change interventions focused on exercise and physical activity behaviour change.

**Table 1:** Summary of the BEEP trial interventions

**Key features**	**Usual physiotherapy care**	**Individually tailored exercise**	**Targeted exercise adherence**
**Number of sessions**	Up to 4 sessions	6 to 8 sessions	8 to 10 sessions
**Time period of treatment**	Up to 12 weeks	Up to 12 weeks	Up to 6 months
**General education**	Advice and information booklet	Advice and information booklet	Advice and information booklet
**Exercise focus**	Focus on lower limb exercise	Focus on lower limb exercise	Focus on both lower limb and general exercise. Signposting and support to engage in general physical activity opportunities in local community
**Individualisation**	Exercises selected from a pre-printed, standardised written template	Exercises individually prescribed for each patient, supported by an individualised, written exercise programme	Exercises individually prescribed for each patient, supported by an individualised, written exercise programme
**Progression**	Minimal progression	Good progression	Good progression
**Supervision**	Minimal supervision	Good supervision	Good supervision
**Exercise monitoring**	No exercise diary	Exercise diary	Exercise and physical activity diaries
**Provision of follow-up**	No follow-up after 12 weeks	No follow-up after 12 weeks	Follow-up and monitoring contacts (telephone or face to face) through to 6 months

All participants may continue to access usual primary care in addition to BEEP treatment. This may include ongoing or new medications, further healthcare consultations with other health professionals, referrals for imaging and surgical opinion and these co-interventions will be recorded on participants’ follow-up questionnaires. For the purposes of the trial, physiotherapy services will contact participants who fail to attend their treatment sessions up to three times in order to try to (re)engage the participant in BEEP treatment sessions. Hydrotherapy, group-based sessions, acupuncture and intra-articular injections will not be permitted in any of the BEEP trial treatment protocols. Intervention fidelity will be assessed through audits of treatment data collected in trial-specific case report forms (comparing these data with the intervention protocol and with the physiotherapy clinical records). We will collect data on the number and content of BEEP treatment sessions using physiotherapy case report forms in all three treatment groups and use those data in our interpretation of the trial results.

### Intervention 1: usual physiotherapy care

Usual physiotherapy care consisting of advice and exercise is the most appropriate control group for the BEEP trial given that randomised trials [15] and systematic reviews [6] consistently show that interventions that include exercise are superior to those which do not. We are not using an attention control group as this trial is designed explicitly as a pragmatic trial, building on evidence about the effectiveness of exercise interventions. Clinical practice guidelines [2] recommend that all patients with OA are advised to exercise and in UK clinical practice, patients are seen in relatively few treatment sessions, and provided with brief relatively standardised exercise programmes that do not take into account differences between individual patients. Our ABC knee study described usual UK physiotherapy practice and formed the basis for the BEEP trial protocol for usual care [14].

The BEEP trial protocol for usual physiotherapy care will consist of advice and lower limb exercise previously tested and shown to be more effective in the short-term than advice alone [15]. Exercises will be selected from an agreed template of commonly prescribed exercises (printed from the commonly used PhysioTools computer software), including specific lower limb muscle strengthening (non-weight-bearing and weight-bearing) and range of movement or stretching exercises. Patients will receive up to 4 one-to-one treatment sessions with a physiotherapist over a period of 12 weeks, during which advice to continue to exercise will be provided but individualisation, progression and supervision of the exercise programme will be minimal, as in usual care. Other interventions used frequently by physiotherapists such as manual therapy and electrotherapy will be permitted as per usual care and they will be recorded on case report forms but the emphasis of the intervention will be supporting the patient to self-care and to follow the advice and exercise programme at home. This usual care protocol matches usual UK practice in that it focuses on lower limb strengthening exercise, relies on self-report rather than the use of exercise diaries to monitor adherence and progress, is delivered over few treatment sessions and thus has limited opportunity for individualisation, supervision or exercise progression and does not offer refresher or booster sessions following the end of the episode of care [14].

### Intervention 2: individually tailored exercise (ITE)

Standardised exercise programmes such as those used in the usual physiotherapy care group in the BEEP trial do not take into account differences between individual patients. Consequently, individuals will be working at relatively different intensities, which for some may be too little to get a training effect, and for others may be too difficult. To be optimally beneficial, exercise should be progressed so that appropriate physical stress is placed upon the individual for further improvements to be obtained [40]. Exercise self-efficacy (confidence to exercise despite the knee pain) has been identified as a predictor of exercise behaviour in many populations [41,42]. Supervision of exercise can enhance patients exercise self-efficacy and self-regulatory skills and reassure them that they can perform the exercises. It also provides good opportunity to reassure the patient about pain responses to specific exercises or to change the exercise prescription to ensure optimal performance or more tolerable pain response. Lack of adequate individual tailoring, supervision and progression of the lower limb exercise programme may in part explain the small benefits seen in some previous exercise trials. Our previous trial results [15,19] suggested that a lower limb exercise programme that was supervised and progressed over six treatment sessions resulted in greater improvements in pain than one provided over an average of four sessions. Therefore the protocol for Individually Tailored Exercise was developed to ensure the prescription of an individualised, supervised and progressed lower limb exercise programme.

The BEEP trial protocol for Individually Tailored Exercise will consist of a supervised individually tailored and progressed exercise programme. The aim of the intervention is to initiate and progress an individually tailored exercise programme, which is supervised in clinic, practiced at home and progressed in terms of intensity over 12 weeks. The intervention is modelled on a previously successful exercise intervention from one of our previous trials [19], and focuses on individualised strengthening (non-weight-bearing and weight-bearing), stretching and balance exercise for lower limb rehabilitation and functional task training. The patient and physiotherapist will define lower limb functional and exercise goals and agree targets that are reviewed and progressed. Individualisation is based on the findings of the physiotherapy assessment of each individual, including biomechanical and physiological observations, pain responses to specific exercises and starting levels of strength, range of movement and balance. Exercises will be prescribed for each individual and participants will be given their own individual print-out of their specific exercise prescription (selected and printed from PhysioTools computer software) and these exercise prescriptions (and print-out instructions) will change over time as the exercise programme is progressed. Physiotherapists will encourage exercise behaviour change using self-monitoring through use of an exercise diary to record their adherence with their lower limb exercise prescription (see Additional file 3). In order to provide greater opportunity for individualisation, supervision and progression of exercise, patients will receive between 6 to 8 one-to-one treatment sessions with a physiotherapist. There will be no scheduled follow-ups (refresher or booster sessions) with the physiotherapist beyond 12 weeks.

### Intervention 3: targeted exercise adherence (TEA)

Adherence to long-term treatment regimes, particularly those involving behavioural components such as exercise, is consistently lower than adherence to medication [43]. Exercise adherence, irrespective of exercise type, may be a key factor determining the success of exercise. In a previous trial of exercise for this patient population, we observed high exercise adherence rates in the short-term through to the end of treatment at 12 weeks, which fell to just over 50% at 12 months [19]. Long-term adherence to lower limb exercise is perhaps unrealistic as these can be challenging to incorporate into daily routines and individual lifestyles and many patients do not consider them enjoyable, stopping them once symptoms reduce or resolve. It may be more realistic to target general physical activities that individuals have previously engaged in and enjoyed (since previous exercise behaviour is theoretically the strongest source of self-efficacy information [41], or physical activities that individuals have positive expectations about (since these positive expectations may increase exercise intentions and ultimately exercise behaviour) in order to support engagement in and adherence to exercise. Our previous ABC knee study [28] highlighted the many different barriers and facilitators to exercise and physical activity, and that no single exercise type or exercise setting is acceptable to all. Thus we designed this Targeted Exercise Adherence intervention to include an adherence-enhancing ‘toolkit’ of optional tools and techniques for physiotherapists to use with different participants, based on their assessment of individual participants and early feedback from participants (See Additional file 4 for summary of contents of the toolkit). The content of the intervention was directly informed by the results of our Cochrane systematic review [26] and a networking meeting, supported by Arthritis Research UK, during which national experts and patient advocates agreed the intervention. Whilst the general physical activity identified and encouraged by physiotherapists will be individualised for participants, we anticipate that many may choose walking as it is seen as inexpensive and accessible. Pedometers have been shown to increase physical activity [44] and to increase step counts in older people [45] and therefore we will include pedometers within the suite of options for physiotherapists to give participants who wish to target increases in walking activity.

The BEEP trial protocol for Targeted Exercise Adherence will begin with a focus on the lower limb (as in the Individually Tailored Exercise group) but transitions to focus increasingly on general physical activity adherence over time. In addition to prescribing an individualised, progressed and supervised lower limb exercise programme, physiotherapists will assess patients’ current general physical activity levels, their intentions to increase their physical activity levels, their attitudes to exercise for knee pain and general health and explore their individual barriers and potential facilitators to exercise. This group will receive 4 treatments up to week 12 and a further 4 to 6 contacts from week 12 through to 6 months (a total of 8 to 10 treatment contacts). In the first 4 treatments, the aim will be for participants to initiate and progress an exercise programme with supervision that will include lower limb and general exercise, and to identify (with the support of the physiotherapist) general physical activity opportunities within the local community that are suitable for, and interest, the individual. Pro-active follow-up from the physiotherapist will take place from week 12 through to 6 months, using choices of telephone and face-to-face contact, providing an additional 4 to 5 contacts with each participant. The aim is to enhance long-term exercise adherence, promote increased general physical activity and encourage participants to develop an exercise ‘habit’, shifting the focus away from lower limb exercise in the earlier treatment sessions and towards sustainable lifestyle changes in physical activity in later treatment sessions. The follow-up sessions will ascertain and promote exercise adherence, increases in physical activity levels, support patients to integrate exercises into their activities of daily living, allow repetition and amendment of the exercise programme based on individuals’ experiences or concerns and progression at a pace suitable for the individual. The adherence enhancing ‘toolkit’ contains different educational and behavioural tools for facilitating physical activity behaviour change, selected for use based on an individualised assessment of each patient. As our Cochrane review identified that no one model of behaviour change was superior for facilitating adherence to exercise for chronic musculoskeletal pain, different theoretical models have underpinned the development of the toolkit, including self-efficacy [42] and self-regulation theory [46]. Tools will include self-monitoring through use of exercise and physical activity diaries (See Additional file 5), SMART goal-setting (specific, measurable, achievable, realistic, time-related) to facilitate the translation of intention into physical activity behaviour, reminders, corrective feedback and reinforcement, behavioural contracting for physical activity [47], pedometers to support increases in walking, example templates to discuss and generate an individual exercise set-back plan, and information about (with active support to access) local physical activity opportunities and facilities in the community. The target by the end of the 6 months period is that participants are engaged in physical activity opportunities within their locality and have had support from the physiotherapist to overcome initial problems or barriers in engaging in these activities. The emphasis is therefore on maintenance of physical activity beyond the period of support from a health professional and NHS-based programme.

### Physiotherapists delivering the interventions

Up to 50 physiotherapists in the participating services will be trained and supported to deliver one of the three BEEP trial interventions. A training programme has been developed and tested in the pilot study and consists of a stepped training course as follows: physiotherapists delivering usual care will attend the first day only, those delivering Individually Tailored Exercise will attend the first day plus a further two days of training (total of three days of training) and those delivering Targeted Exercise Adherence will attend the first three days plus a further two days (total of five days of training). On-going support to adhere to the treatment protocols will be offered through PCRN physiotherapy research facilitators who have also undertaken the BEEP training programme and who will conduct regular audits of physiotherapy case report forms to investigate treatment fidelity. All physiotherapists will be invited to evaluate the training programme through completion of brief questionnaires before and directly following their training and at the end of treatment of all BEEP trial participants (approximately 18 months later). Further workshop refresher sessions to support BEEP physiotherapists will be provided during the course of the trial. The full details of the content and justification for the training programme and the observed changes in physiotherapists’ attitudes, beliefs and intended behaviours through the use of case vignettes will be provided in full in a separate publication.

### Outcome measures

#### Primary outcomes

This trial has two primary outcomes, lower limb pain and function measured using the Western Ontario and McMaster Universities Osteoarthritis Index (WOMAC) [48], collected at baseline and all follow-up time-points (3, 6, 9, 18 and 36 months). The primary time-point is 6 months after randomisation. The psychometric properties of the WOMAC [48] have been extensively studied in knee pain populations in clinical trials of different interventions including exercise [49] and the WOMAC has been recently shown to be the most responsive of five pain measures [50]. The pain subscale ranges from 0 (no pain) to 20 (maximum pain) and the function subscale ranges from 0 (no disability) to 68 (maximum disability). It is particularly suitable for the BEEP trial as it specifically captures self-reported pain during activities and the degree of difficulty with everyday physical activities, both of which are key treatment targets of physiotherapy-led exercise.

#### Secondary outcomes

A range of secondary outcomes will be collected: the proportion of treatment responders using the internationally agreed Outcome Measures in Rheumatology Clinical Trials (OMERACT-OARSI) clinical responder criteria [16,17] that combines data on pain and function from the WOMAC [48] with patient’s global assessment of change (recorded using a 6 point Likert Scale); physical activity levels (Physical Activity Scale for the Elderly (PASE) which assesses physical activity levels over a 1-week period combining physical activity from several domains including household, occupational and leisure [51]); self-reported body mass index (calculated from self-reported height and weight); exercise adherence (attendance at treatment sessions, self-reported adherence to prescribed exercise programme); use of local physical activity facilities in the previous 7 days (single item); a modified version of a measure of treatment acceptability and credibility [52,53]; a measure of illness perceptions (Brief Illness Perceptions Questionnaire [54]); confidence in ability to exercise (Self-efficacy for Exercise Scale [55]); outcome expectations from exercise (Outcome Expectations for Exercise Scale 2 [56]); anxiety (Generalised Anxiety Disorder Assessment 7 [57]); depression (Personal Health Questionnaire Depression Scale [58]); self-reported health care resource use (both NHS and private health care); and overall health status (EQ-5D-3 L [59]). Resource use and EQ-5D-3 L data will be used in the cost-utility analysis (further details are provided in the ‘Economic analysis’ section).

#### Accelerometry outcomes

Physical activity will also be measured in a subsample of participants (a target of n = 30 from each group), through snap-shots of 7-day accelerometry at each follow-up time-point. Accelerometers are motion sensors worn on the hip and will be used in this trial to estimate physical activity as counts per minute, time spent in light, moderate and vigorous physical activity and proportions of people who meet guideline levels of physical activity [60]. Accelerometers will be allocated at the point of randomisation. The accelerometer units will be posted out to the participants with full instructions. Participants will be asked to wear the unit during waking hours for 7 consecutive days and then to post the unit back to the research centre where the data collected will be downloaded and analysed. During the trial, accelerometers will be allocated at regular intervals (approximately monthly) to the next three participants randomised to each treatment group. A random allocation procedure will not be used, as not all participants will be willing to wear them. A regular accelerometer allocation procedure is needed to enable accelerometers to be available to collect data for all 90 participants (at baseline and follow-up) from a pool of 30 accelerometers available for the trial. The allocation procedure will be phased in at the start of the trial to ensure that the system is running smoothly and that accelerometers are being returned in time to be reused by other participants. Those who do not return their accelerometers will be posted a written reminder.

#### Follow-up

Outcome measures will be collected by self-report, postal questionnaire before randomisation and at 3, 6, 9, 18 and 36 months (Figure 1). Therefore, adherence to exercise will be measured twice throughout the early phase (0 to 6 months) and three times during the maintenance phase of the exercise interventions (9 to 36 months). Non-responders will be followed up using our standardised CTU follow-up procedures for trials. This comprises a questionnaire, a reminder postcard at two weeks and a further copy of the questionnaire at four weeks. For participants who do not respond to any of these reminders, we will aim to collect minimum outcome measure data via telephone (by research nurses who are blind to treatment allocation) and post at key outcome time-points (6 months, 18 months and 36 months) in order to try to capture primary outcome data and to minimise missing data. Quality assurance processes within the CTU will ensure training and auditing of the research nurses conducting minimum data telephone calls. In order to reduce participant burden the 3 and 9 month questionnaires will be slightly shorter than those at 6, 18 and 36 months and as direct measures of physical activity may themselves increase physical activity similar measures of physical activity will be used in each intervention group. Table 2 includes a list of all measures and the time-points at which they will be collected.

**Table 2:** Outcome measures

**Data collection**	**Measurement scale**	**Time points (months)**
*Participant characteristics*		
Age	Years	0,3,6,9,18,36
Gender	Female/Male	0,3,6,9,18,36
Weight	Stones and lbs or Kilograms	0,3,6,9,18,36
Height	Feet and inches or centimeters	0
Marital status	Married/separated/divorced/widowed/cohabiting/single	0
*Work factors*		
Current/most recent job title	Free Text	0,6,18,36
Currently in a paid Job	Yes/No	0,6,18,36
Working hours	Working full time (30 hours or more per week)/working part time (29 hours or less per week)/	6,18,36
Time off because of knee pain including time off to visit any health care professional	Yes/No (during last 6 months)	6,18,36
How many days weeks, weeks or months were you absent from work due to knee problem	Number of days/weeks/months (during last 6 months)	6,18,36
*Knee Problem*		
Knee with most discomfort	Tick boxes for left or right	0,3,6,9,18,36
Duration of knee problem	In the last 12 months/More than 1 year but less than 5 years ago/More than 5 years but less than 10 years ago/I have had this knee problem for more than 10 years	0
Global assessment of change in knee problem	Completely recovered/much better/better/no change/worse/much worse (since first seen by the physiotherapist)	3,6,9,18,36
Knee pain^α^	0–20	0,3,6,9,18,36
Stiffness^α^	0-8	
Function^α^	0 - 68	
	(WOMAC [48])	
Illness perceptions^α^		0,3,6,
- Consequences	0-10	
- Timeline	0-10	
- Personal control	0-10	
- Treatment control	0-10	
- Identity	0-10	
- Concern	0-10	
- Understanding	0-10	
- Emotional response	0-10	
	(IPQ-brief modified for knee pain [54])	
*Physical Activity & Exercise*		
Experience of exercise	Personal experiences of exercise	0
Use of local facilities^α^	Use of local facilities for physical activity in the last 7 days	0,3,6,9,18,36
Self-efficacy for exercise^α^	0-10 (SSE scale [55])	0,3,6
Outcome expectations for exercise^α^	1-5 (OEE-2 [56])	0,3,6
Physical activity^α^	0-400+ (PASE [51])	0,3,6,9,18,36
Exercise adherence and treatment credibility	Confidence in and adherence to treatment plan	3,6,9,18,36
Accelerometry^α^	Average counts per minute	For a sub-sample of participants 0,3,6,9,18,36
	Meeting physical activity guidelines [60]	
*General health and well being*		
Depression^α^	0-24 (PHQ8 [58])	0,3,6,18,36
Anxiety^α^	0-21 (GAD7 [57])	0,3,6,18,36
Quality of life^α^	-0.59-1 (EQ-5D-3 L [59])	0,3,6,9,18,36
Body manikin (pain)	Body area shaded to represent pain last a day or longer in the last 4 weeks	0
Co-morbidity	Tick boxes for key co-morbidities	0
*Managing your knee problem*		
Current medication for knee problem	Prescribed and over the counter medications (with dosage and length of supply)	0,3
Prescribed medication for knee problem	Prescribed medications in the last 6 months/12 months/18 months (with dosage and length of supply)	6,18,36
Cost of over the counter treatments/appliances	e.g. painkillers, anti-inflammatory drugs, TENS machine, hot and cold packs, knee supports (£ in the last 6 months/12 months/18 months)	6,18,36
Costs for use of local facilities/opportunities involving physical activity	(£ in the last 6 months/12 months/18 months)	6,18,36
Healthcare Utilisation	Contact with NHS and private healthcare professionals, number of visits/inpatient stays, types of investigations/treatments in the last 6 months/12 months/18 months	6,18,36

Key: EQ-5D-3 L = Quality of Life; GAD 7 = Generalized Anxiety Disorder 7; IPQ – Brief = The Brief Illness Perception Questionnaire; OEE-2 = Outcome Expectations for Exercise Scale - 2; PASE = Physical Activity Scale for the Elderly; PHQ8 = Patient Health Questionnaire 8; SEE = Self-Efficacy for Exercise Scale; WOMAC = Western Ontario and McMaster Universities Arthritis Index. α = Measure used as an outcome to test for clinical effectiveness (along with Body mass index (BMI) and the OMERACT-OARSI responder criteria [16,17], which are not indicated on this table as derived by combining individual measures of height and weight for BMI and WOMAC pain and function with global assessment of change for the OMERACT-OARSI responder criteria).

#### Adverse events

The occurrence of adverse events from all interventions will be monitored and assessed using case report forms, contact with the trial co-ordinator, physiotherapist report, and follow-up questionnaires. A common adverse event from unaccustomed exercise and physical activity is temporary, mild muscle soreness. Physiotherapists delivering the interventions will advise participants about how to manage such symptoms.

Each physiotherapy site will report any serious adverse event (SAE) experienced by a trial participant immediately to the trial Chief Investigator that may possibly be related to either the interventions or the trial procedures. The Chief Investigator will assess whether the event was related to or resulted from any of the BEEP trial interventions or procedures, according to the process laid out in the BEEP trial Standard Operating Procedure for SAEs. Any SAE considered to be related to the trial procedures or interventions will be reported to the main Research Ethics Committee by the Chief Investigator within 15 days of her becoming aware of the event. In addition, all such events will be reported to the trial sponsor, Trial Steering Committee and Data Monitoring Committee.

#### Other data

Other variables collected in participant questionnaires are age, gender, marital status, co-morbidities, pain location using a pain manikin, duration of the knee problem, experience of exercise and work status.

The following process data will also be recorded from the physiotherapy case report forms: number of treatment sessions attended, the main content of each treatment, physiotherapists contact time with participants, the number of treatment withdrawals and treatment non-attendances (DNAs).

### Sample size

The sample size for the main trial will be based on the primary outcome measures: WOMAC pain and function subscales [48] and will compare 6 months post-randomisation outcome for the usual care group with either of the two other intervention groups. We chose 6 months since our TOPIK trial [15] showed the effects of exercise had reduced by this point with usual physiotherapy care. The BEEP trial is powered to detect an effect size of 0.35 for both WOMAC pain and function; an effect size classified as ‘small’ to ‘moderate’ using standard benchmarks by Cohen [61]. We chose to power the trial based on a specified effect size (rather than minimum important change) as Terwee et al 2009 [62] report a lack of consensus as to minimum important change on the WOMAC. From our previous trials [19], we estimate that the standard deviation for WOMAC pain and WOMAC function at 6 months follow-up will be 5 and 17 respectively. An effect size of 0.35 therefore equates to a 1.75 point difference on WOMAC pain and a 5.95 point difference on WOMAC function. To achieve 80% power and a 5% significance level (two tailed) we require 129 patients per treatment group, giving a total sample of 387 patients [63]. Allowing for a 20% loss to follow-up rate (informed by our previous knee pain trials [15,19]) we aim to randomise a total of 500 participants to the trial over a period of 18 months.

For practical reasons, the sample size will not be inflated to allow for clustering of individual patients being treated by the same physiotherapist [64,65], but rather the trial will provide useful estimates of clustering effects and we will adjust for therapists in a sensitivity analysis. It is anticipated that a minimum of 36 physiotherapists (12 per treatment group) will be trained to deliver the trial treatments and that each physiotherapist will treat approximately 18 patients. Thus at the end of the trial, we will be able to estimate an intra-class correlation (ICC) to inform future similar trials.

A formal power calculation was not completed to determine the number of patients to wear an accelerometer. The sample size of 30 per group is based on practical considerations (restricted by the number of accelerometers available for data collection) and on having a reasonable sample size to analyse the data using parametric statistics.

### Embedded qualitative interviews

Qualitative methods have an important role to play in evaluating complex interventions [66] and in helping to interpret the findings from RCTs [67,68]. In the BEEP trial embedded, longitudinal, qualitative interviews will explore participants’ experiences of treatment, their views of the acceptability of each intervention, the impact of the interventions on participants' exercise and general physical activity behaviour and the explanations for change in knee symptoms and exercise behaviour over time. Topic guides will include questions that explore participants’ views of their physiotherapy treatment, how they got on with their exercise programme and what factors they felt helped or hindered them to adhere to the exercises following the end of their treatment contact with the physiotherapist, and approximately 12 to 18 months later. We will also ask what they feel the future holds for their knee problem. Interviews will be semi-structured, face to face and will last up to one hour. Interviews will be longitudinal in that they will be conducted following physiotherapy treatment completion, and also in the longer-term 12 to 18 months later (timed to be after their 18 month BEEP follow-up questionnaire). The number invited will be determined by ongoing data analysis and theme saturation but it is anticipated to continue until approximately 30 sets of longitudinal interviews have been conducted. Purposive sampling based on data collected in the 3 and 6 months follow-up questionnaires will ensure a diverse range of characteristics in terms of demographic details (age and gender), intervention group, severity of knee condition determined by WOMAC pain and function scores (as this could be linked to participants’ willingness to exercise), and changes in WOMAC scores after intervention completion (as these could be linked to participants’ views about success of the BEEP interventions and longer term exercise adherence). With participants’ written consent, interviews will be audio-recorded and transcribed verbatim by professional transcribers. All transcripts will be anonymised and data management and analysis will be facilitated by NVivo (QSR International, Version 9). Earlier interviews (at the end of physiotherapy treatment completion) will be analysed and inform the interview content of the interviews at the longer-term follow-up.

### Statistical analyses

The trial analysis will be conducted and reported using the Consolidated Standards of Reporting Trials (CONSORT) guidelines [65,69,70].

#### Recruitment and follow-up

The number of participants identified and recruited using each recruitment method will be reported in a flow chart, along with the number of participants returning a questionnaire at each follow-up stage. The flow chart will include reasons for trial ineligibility and withdrawal (when available) and will be used to compare the recruitment success of each method to inform future trial recruitment.

The baseline characteristics of participants will be reported for the three recruitment methods, for the three treatment groups (to explore the effectiveness of randomisation) and for those with and without data at each follow-up time point (to explore any selective loss to follow-up). Any differences between treatment groups will be described by visual inspection rather than statistical testing.

#### Primary and secondary trial analysis for clinical outcomes

The primary and secondary trial analysis will be conducted blind to intervention group by the statistician (EN) and an independent statistician will verify the analysis of the primary outcomes. The primary analysis will compare each of the Individually Tailored Exercise and Targeted Exercise Adherence interventions to usual physiotherapy care on an intention-to-treat basis, for the primary outcome at 6 months post-randomisation. Secondary analysis will include the analysis of the primary outcome at the secondary endpoints (3, 9, 18 and 36 months) and analysis of the secondary clinical effectiveness outcomes, as indicated in Table 2, at all follow-up time points. Estimates of clinical effect will be derived using analysis of covariance (ANCOVA) for continuous outcome measures and logistic regression for dichotomous outcomes and will be presented as mean or percentage differences (as appropriate) with 95% confidence intervals after adjustment for baseline covariates defined *a priori* as:

• Baseline for the outcome of interest^a^

• Age

• Gender

• Duration of the knee problem

• Physiotherapy treatment centre (as was used in the randomisation algorithm)

A secondary analysis will also be completed to model the longitudinal trajectory of WOMAC pain and function over time (the primary outcome) and the PASE physical activity measure using generalized estimating equations. Model predictors will include the *a priori* covariates listed above (with the baseline for the outcome of interest modelled as a covariate rather than outcome as recommended in Peduzzi et al [71]), time, treatment and a time*treatment interaction. A linear model will be fitted to the data (with time as a continuous measure) initially, however, if the trajectory over time is non-linear, quadratic or cubic terms will be explored. It will also be explored that conclusions do not differ if time is represented in the model as a categorical variable. All model estimates will be presented with 95% confidence intervals derived using robust standard errors.

The primary and secondary trial analyses will be conducted after imputation of missing data has been completed. Missing data will be imputed using the multiple imputation routines in STATA v11. All primary and secondary clinical effectiveness outcomes (excluding accelerometry) and the a priori covariates will be included in the imputation model and will have their missing data imputed. The intention-to-treat analysis will include participants who are protocol violators, but will not include participants who are post-randomisation exclusions. Information on any adverse events will be reported. We plan to analyse and publish the results of the trial after the 18 month follow-up is complete, and then produce a follow-up paper at 36 months.

#### Sensitivity analysis for clinical outcomes

The following sensitivity analyses will be completed and reported if they change the interpretation of the findings from the main analysis described above.

• *Therapist effects:* these will be explored by adding a random effect term (to represent the treating therapist) to the main treatment models. The models with and without the therapist effect will be compared at each outcome time-point to explore the impact of the treating therapist on the effect estimates obtained.

• *Imputation of missing data:* a complete case analysis will be conducted and the results from this compared to those from imputed data.

• *Model covariates:* treatment models will be run on an unadjusted basis to investigate if the inclusion of model covariates has an impact on the main trial findings.

• *A per protocol analysis:* this will be completed on the subsample of participants receiving treatment in line with the specified treatment protocols. Criteria for determining the per protocol group assignment will be established by the Trial Management group and approved by the Trial Steering Committee before analysis begins.

#### Exploratory subgroup analysis

A set of exploratory analyses will investigate the factors that moderate the effects of exercise in participants with knee pain. Models will be derived using WOMAC pain and function as the dependent variables, and intervention group and other potentially important factors (demographic, knee-specific, exercise adherence, general health, attitudes and outcome expectations for exercise will be put forward as candidate independent variables). A specific subgroup analysis with strong theoretical rationale will compare the clinical outcomes of pain and function of those who report high exercise adherence across all intervention groups (versus those who report lower exercise adherence). We hypothesise that those who report higher adherence will have improved pain and function outcomes.

#### Analysis of adherence data

Descriptive statistics will be used to examine exercise adherence and will be reported as numbers and percentages, or means and standard deviations, as appropriate. Exercise adherence will be measured by reporting the number of treatment sessions attended in each treatment group, and by reporting the following measures at each follow-up time-point:

• Self-reported exercise adherence (measured by agreement with the statement ‘I have been doing my exercises as often as I was advised’)

• Frequency and duration of physiotherapy exercise completed by the participant

• Change (from baseline) in level of physical activity (measured by PASE and, in a subsample of participants, accelerometry)

• Use of local exercise and physical activity facilities

#### Analysis of accelerometer data

Accelerometry data from the subsample of BEEP trial participants will be analysed using ActiGraph (version 6) accelerometer software. Prior to analysis, data will be cleaned by excluding time periods containing more than 60 minutes of zero count (where it is assumed the accelerometer is not being worn) and by including only those participants who have worn the accelerometer for at least 5 days for 10 hours or more. Sensitivity analyses will be conducted by changing the threshold to exclude minutes of zero count from 60, to 30 and 90 minutes respectively with the later threshold recommended for participants with knee pain [72]. For each participant we will generate the following at each data collection point: number of valid minutes, counts per minute, proportion of time spent at each level of physical activity, using the cut-offs from Freedson et al [73]. We will also calculate the proportion of participants meeting exercise/physical activity recommendations [60].

Descriptive statistics will be given for the accelerometer variables at baseline and by treatment arm. Change (between baseline and each follow-up time point) in the average count per minute and in the proportion meeting the exercise recommendations will be analysed using analysis of covariance (ANCOVA) and logistic regression respectively with analyses adjusted for the covariates defined for the clinical effectiveness analysis and by treatment arm.

### Economic analysis

The economic evaluation will determine the cost-effectiveness of the two new physiotherapy-led exercise interventions to improve individual tailoring of, and adherence to, exercise in knee OA patients in primary care, in comparison to usual physiotherapy care. A cost-consequence analysis will initially be reported, describing all the important results relating to costs and consequences. Subsequently, an incremental cost-utility analysis will also be undertaken using patient responses to the EQ-5D-3 L questionnaire to calculate the incremental cost per additional quality-adjusted life year (QALY) gained.

#### Costs

Information on resource use and time off work will be collected from patient-completed questionnaires at 6 months, 18 months and 36 months. Health sector costs include primary and secondary care contacts, investigations, pain medications and contacts with other health care professionals. The cost of the interventions will also be determined taking into account any additional resource use required to deliver those interventions such as additional physiotherapy visits and telephone contacts and any additional equipment (e.g. pedometers) supplied to patients. The cost of the advice and information booklet will not be included as this will be given to all trial participants and will therefore be cancelled out in the cost analysis. Questions on patients’ personal expenditure will concentrate on private health care use and over-the-counter treatments. Due to the lack of nationally representative unit cost estimates for private health care, this care will be costed as the NHS equivalent in the base-case. Patient reported costs for over-the-counter treatments will be used.

Resource use will be multiplied by unit costs obtained from standard sources and health care providers including the British National Formulary (BNF), Unit Costs of Health and Social Care and NHS Reference costs [74-76]. Productivity costs will be calculated using data collected on employment status at every time point and days off work due to knee pain. For those in paid employment, information on occupation, further details of typical work activities and the nature of their employment (full time or part time) will be requested. The average wage for each respondent will be identified using UK Standard Occupational Classification coding [77] and annual earnings data for each job type [78]. The analysis will use the human capital approach, and the self-reported days of absence will be multiplied by the respondent-specific wage rate.

The data for costs are likely to have a skewed distribution therefore the plan is to explore the nature of the distribution of costs. If the data are not normally distributed, then a non-parametric comparison of means, using bootstrapping, will be undertaken [79].

#### Health economic outcomes

All patients will be asked to complete the EQ-5D-3 L questionnaire at baseline and at 3, 6, 9, 18 and 36 months in order that quality-adjusted life years (QALYs) over the time period can be calculated for each study participant, using the area under the curve method. If differences in baseline characteristics occur between intervention groups, for example in baseline EQ-5D-3 L score, the analysis will control for these baseline differences using regression-based adjustment [80].

#### Cost-effectiveness analysis

The primary economic analysis will be conducted at 18 months, with additional analysis at 36 months. An intention-to-treat analysis will be undertaken. Multiple imputation techniques will be used to deal with missing EQ-5D-3 L scores and resource use data, ensuring that all eligible trial participants are included in the base case economic evaluation. All estimates will be presented as means with 95% confidence intervals.

The base case cost analysis will adopt a NHS and personal social services (PSS) perspective. A broader costing perspective will be considered in a sensitivity analysis, taking into account NHS/PSS costs, patients’ personal expenditure and costs associated with work loss. All costs and outcomes beyond 12 months will be discounted at the standard UK rate of 3.5%.

The estimation of cost-effectiveness within this trial will focus on the principles of dominance and extended dominance. Dominance is a straightforward concept; if an intervention is less effective and more costly than at least one of its comparators, it is not included for further consideration with regard to the estimation of cost-effectiveness. Extended dominance is applied in incremental cost-effectiveness analysis when an intervention is less effective and more costly than a linear combination of two other strategies; the purpose is to remove from consideration those strategies whose costs and benefits are improved by a mixed strategy of two other alternatives. The practical application of cost-effectiveness analysis is to compare an intervention with the next most effective strategy, therefore strategies are ordered in term of QALYs from least to greatest. Failure to remove all dominated or extendedly dominated strategies may lead to comparisons that are not with the next best alternative but with irrelevant alternatives.

The robustness of the results will be explored using sensitivity analysis. Deterministic sensitivity analysis will explore uncertainties in the methods employed to analyse the data, for example a complete case analysis as an alternative to using an imputed data set, any assumptions made in the analysis and the generalisability of the results to other settings.

Probabilistic sensitivity analysis (PSA) will explore the uncertainty in the trial based data itself. Bootstrapping will be undertaken using STATA to produce 5000 bootstrap replications of cost-QALY difference pairs. These will be plotted on a cost-effectiveness plane to illustrate the uncertainty in the confidence to be placed on the results of the economic analysis. This will be further explored by estimating cost-effectiveness acceptability curves (CEACs) [81]. These plot the probability that the intervention is cost-effective against threshold values for cost-effectiveness.

Finally, if one or more of the interventions demonstrates effectiveness for the full duration of the trial, the longer-term costs and benefits associated with that intervention will be explored using decision modelling approaches to extrapolate beyond the trial follow-up.

### Qualitative analyses

Data collection and analysis will be carried out iteratively so that emerging themes in the analysis can be explored in depth in the subsequent interviews. Earlier interviews (at the end of physiotherapy treatment completion) with individual participants will be analysed and inform the interview content of the interviews at the longer-term follow-ups. Sampling will continue until no new themes emerge.

We will use an emergent and layered approach to analysis [82] that will allow for both induction and deduction. First, using the principles of constant comparison [83], we will open code all transcripts. This will enable patient experiences of the interventions, general views on exercise and barriers and facilitators to exercise to be explored. A researcher will code the transcribed data, with a sample of interviews being independently coded by other members of the research team to ensure transparency and agree emergent themes at successive stages of the data collection and analysis. Second, we will apply a more deductive approach by re-reading transcripts and allocating data to predetermined codes of individualisation, supervision and progression (three core intervention constructs). Third, a focused within-case and cross-case longitudinal analysis will be performed by asking descriptive and interpretative questions of the data [84]. Descriptive questions will investigate what has changed in terms of participants’ knee condition, adherence to the prescribed exercise programme or general physical activity levels, and the key influences on these changes. This will enable an overall understanding of what is different from the first interviews (post intervention) to the second (12 to 18 months after the end of the BEEP trial intervention) and potential reasons for any changes. Interpretive questions will then investigate, for example, which changes interrelate and how these might relate to existing theories of exercise adherence. Data summary frameworks [82,84] will facilitate identifying patterns of change across time. Qualitative data analysis will be undertaken separate to the quantitative data analysis in the first instance to facilitate an interpretative approach and not constrain the analysis by quantitative variables or findings.

### Trial organisation and monitoring

The BEEP trial is sponsored by Keele University. The day to day operation of the trial will be overseen by a Trial Management Group (led by NEF) and the trial will be monitored by an independent Trial Steering Committee (TSC), chaired by Prof Michael Hurley. The TSC will be made up of individuals with expertise in musculoskeletal research, exercise physiology and rheumatology. The committee will include two lay members with osteoarthritis. An independent Data Monitoring Committee (DMC) will also monitor the study, chaired by Prof Chris Roberts. Terms of reference for the DMC and TSC are available on request from the BEEP trial team. During the trial period through to 18 months follow-up, no interim analyses are planned, unless judged necessary by the DMC. The TSC, DMC, Trial Management Group and clinical partners will remain ignorant of the trial results until the 18 month follow-up data time-point.

### Data confidentiality and archiving

All trial-related information will be stored securely at the Arthritis Research UK Primary Care Centre at Keele University. Data will be anonymised using coded identification numbers to depersonalise data with the housing of the data and the linking code in separate locations, under password protection. Access to the data will be to the small number of individuals necessary for quality control, audit and analysis. The final trial dataset will be accessed by the statistician and the trial principal investigator (NEF). We will publish and communicate the trial results regardless of the outcome of the trial. Data from the BEEP trial will be archived and made available for future, secondary analysis and data pooling purposes from the Arthritis Research UK Primary Care Centre at Keele University.

### Ethical review

The trial received research ethical approved by the North West 1 Research Ethics Committee, Cheshire, UK (REC reference: 10/H1017/45) and site-specific approvals have been received from the appropriate local research and development offices, and we will provide annual reports of progress. The trial is being conducted in accordance with the ethical principles in the Declaration of Helsinki and good practice guidelines on the proper conduct of research.

## Discussion

The BEEP trial will compare the clinical and cost-effectiveness of three physiotherapist-led, exercise-based interventions, for older adults with knee pain attributable to OA in primary care. In comparison to existing trials of exercise for knee OA, the strengths of the BEEP trial are its size, long-term follow-up, and inclusion of a cost-effectiveness analysis and longitudinal qualitative interviews with participants. The main limitation, common to many trials of non-pharmacological interventions, is the inability to blind participants to treatment allocation.

The BEEP trial will inform GPs, physiotherapists, NHS managers and service commissioners about how to optimise the primary care management of older adults with knee pain and about the resources needed to achieve it. It will directly inform this patient group how to optimise the benefits from exercise, inform Primary Care and physiotherapy services about the effectiveness of their management of knee pain in older adults and inform future education of healthcare practitioners. It may also help to delay and prevent some individuals from becoming surgical candidates.

## Endnotes

^a^Baseline adjustment is not relevant for the OARSI responder criteria as it incorporates baseline levels of knee pain and function into the follow-up outcome.

## Abbreviations

ABC knee: Attitudes and behaviours concerning knee pain; ANCOVA: Analysis of covariance; APEX: Acupuncture, Physiotherapy and Exercise; BEEP: Benefits of Effective Exercise for Knee Pain; BNF: British National Formulary; CEAC: Cost-Effectiveness Acceptability Curve; CONSORT: Consolidated Standards of Reporting Trials; CTU: Clinical Trials Unit; DNA: Treatment Non-attendance; DMC: Data Monitoring Committee; GP: General Practitioner; ICC: Intra-class correlation; ITE: Individually Tailored Exercise; NHS: National Health Service; NICE: National Institute for Health and Clinical Excellence; NIHR: National Institute for Health and Clinical Excellence; OA: Osteoarthritis; OARSI: Osteoarthritis Research Society International; OMERACT: Outcome Measures in Rheumatology Clinical Trials; PASE: Physical Activity Scale for the Elderly; PCRN: Primary Care Research Network; PCT: Primary Care Trust; PIS: Participant information sheet; PSA: Probabilistic Sensitivity Analysis; PSS: Personal Social Services; QUALY: Quality Adjusted Life Year; RCT: Randomised Controlled Trial; REC: Research Ethics Committee; SAE: Serious Adverse Event; SMART: Specific, measurable, achievable, realistic, time-related; TEA: Targeted Exercise Adherence; TOPIK: Treatment Options for Pain in the Knee; TSC: Trials Steering Committeel; UC: Usual care; UK: United Kingdom; WOMAC: Western Ontario and McMaster Universities Index.

## Competing interests

The authors declare that they have no competing interests.

## Authors’ contributions

NEF and EMH conceived of the trial, developed the trial design and secured funding. NEF is the chief investigator, leads the trial management team, and produced the first draft of the BEEP trial protocol. ELH, MAH and ER contributed to the development of the trial design, interventions and the physiotherapist training programme. EN provided statistical expertise and developed the detailed statistical analysis plan for the BEEP trial. DGTW was the lead economist at the conception of the trial and was responsible for designing the economic evaluation, including the selection of outcomes and design of the self-report questionnaires. SJ contributed to the economic evaluation plan and is overall lead for the health economic analysis. CJ contributed to the design of the qualitative research (with NEF and MAH) and led the analysis plan for the interviews. All authors contributed to the refinement of the detailed trial protocol, drafting and approval of the final manuscript.

## Pre-publication history

The pre-publication history for this paper can be accessed here:

http://www.biomedcentral.com/1471-2474/15/254/prepub

## Supplementary Material

Additional file 1 BEEP trial participant information leaflet. Click here for file

Additional file 2 Copy of advice booklet provided to all BEEP participants. Click here for file

Additional file 3 Copy of lower limb exercise diary. Click here for file

Additional file 4 Summary of contents of adherence enhancing tool kit. Click here for file

Additional file 5 Copy of physical activity diary. Click here for file

## References

[B1] Peat G McCarney R Croft P Knee pain and osteoarthritis in older adults: a review of community burden and current use of primary health care Ann Rheum Dis 2001 60 91 97 11156538 10.1136/ard.60.2.91 PMC1753462

[B2] National Institute for Health and Care Excellence Osteoarthritis: Care and Management in adults. Clinical guideline CG177 National Clinical Guideline Centre 2014

[B3] Duncan RC Hay EM Saklatvala J Croft PR Prevalence of radiographic osteoarthritis: it all depends on your point of view Rheumatology 2006 45 757 760 16418199 10.1093/rheumatology/kei270

[B4] Cibere J Do we need radiographs to diagnose osteoarthritis? Best Pract Res Clin Rheumatol 2006 20 27 38 16483905 10.1016/j.berh.2005.08.001

[B5] Juhl C Christensen R Roos EM Zhang W Lund H Impact of exercise type and dose on pain and disability in knee osteoarthritis Arthritis Rheum 2014 66 622 636 10.1002/art.38290 24574223

[B6] Fransen M McConnell S Exercise for osteoarthritis of the knee Cochrane Database Syst Rev 2008 4 CD004376 18843657 10.1002/14651858.CD004376.pub2

[B7] Smidt N de Vet HC Bouter LM Dekker J Arendzen JH de Bie RA Bierma-Zeinstra SM Helders PJ Keus SH Kwakkel G Lenssen T Oostendorp RA Ostelo RW Reijman M Terwee CB Theunissen C Thomas S van Baar ME van 't Hul A van Peppen RP Verhagen A van der Windt DA Exercise Therapy Group Effectiveness of exercise therapy: a best evidence summary of systematic reviews Aust J Physiother 2005 51 71 85 15924510 10.1016/s0004-9514(05)70036-2

[B8] Taylor NF Dodd KJ Shields N Bruder A Therapeutic exercise in physiotherapy practice is beneficial: a summary of systematic reviews 2002-2005 Aust J Physiother 2007 53 7 16 17326734 10.1016/s0004-9514(07)70057-0

[B9] Uthman OA van der Windt DA Jordan JL Dziedzic KS Healey EL Peat GM Foster NE Exercise for lower limb osteoarthritis: systematic review incorporating trial sequential analysis and network meta-analysis BMJ 2013 347 f5555 24055922 10.1136/bmj.f5555 PMC3779121

[B10] Hurley MV Muscle dysfunction and effective rehabilitation of knee osteoarthritis: what we know and what we need to find out Arthritis Rheum 2003 49 444 452 12794802 10.1002/art.11053

[B11] Van Baar ME Assendelft WJJ Dekker J Oostendorp RA Bijilsma JW Effectiveness of exercise therapy in patients with osteoarthritis of the hip or knee Arthritis Rheum 1999 42 1361 1369 10403263 10.1002/1529-0131(199907)42:7<1361::AID-ANR9>3.0.CO;2-9

[B12] Hurley M Dziedzic K Bearne L Sim J Bury T The clinical and cost effectiveness of physiotherapy in the management of elderly people with common rheumatological conditions: evidence briefing 2002 London: Chartered Society of Physiotherapy http://csplis.csp.org.uk/webview/?infile=details.glu&loid=33508

[B13] Puett DW Griffin MR Published trials of non-medicinal and non-invasive therapies for hip and knee osteoarthritis Ann Intern Med 1994 121 133 140 8017727 10.7326/0003-4819-121-2-199407150-00010

[B14] Holden MA Nicholls EE Young J Hay EM Foster NE Physical therapists’ use of therapeutic exercise for patients with clinical knee osteoarthritis in the United Kingdom: in line with current recommendations? Phys Ther 2008 88 1109 1121 18703675 10.2522/ptj.20080077 PMC2557052

[B15] Hay EM Foster NE Thomas E Peat G Phelan M Yates HE Blenkinsopp A Sim J Pragmatic randomized clinical trial of the effectiveness of community physiotherapy and enhanced pharmacy review for knee pain in older people presenting to primary care BMJ 2006 333 995 998 17056608 10.1136/bmj.38977.590752.0B PMC1635605

[B16] Pham T Van Der Heijde D Lassere M Altman RD Anderson JJ Bellamy N Hochberg M Simon L Strand V Woodworth T Dougados M OMERACT-OARSI Outcome variables for osteoarthritis clinical trials: The OMERACT-OARSI set of responder criteria J Rheumatol 2003 30 1648 1654 12858473

[B17] Pham T van der Heijde D Altman RD Anderson JJ Bellamy N Hochberg M Simon L Strand V Woodworth T Dougados M OMERACT-OARSI initiative: Osteoarthritis Research Society International set of responder criteria for osteoarthritis clinical trials revisited Osteoarthritis Cartilage 2004 12 389 399 15094138 10.1016/j.joca.2004.02.001

[B18] Hay E Barlas P Foster N Hill J Thomas E Young J Is acupuncture a useful adjunct to physiotherapy for older adults with knee pain?: the "acupuncture, physiotherapy and exercise" (APEX) study BMC Musculoskelet Disord 2004 2 31 15345098 10.1186/1471-2474-5-31 PMC520743

[B19] Foster NE Thomas E Barlas P Hill JC Young J Mason E Hay EM Acupuncture as an adjunct to exercise based physiotherapy for osteoarthritis of the knee: randomised controlled trial BMJ 2007 335 436 17699546 10.1136/bmj.39280.509803.BE PMC1962890

[B20] Sullivan T Allegrante JP Peterson MGE Kovar PA MacKenzie CR One-year follow up of patients with osteoarthritis of the knee who participated in a program of supervised fitness walking and supportive patient education Arthritis Care Res 1998 11 228 233 9791321 10.1002/art.1790110403

[B21] Thomas KS Muir KR Doherty M Jones AC O'Reilly SC Bassey EJ Home based exercise programme for knee pain and knee osteoarthritis: randomised controlled trial BMJ 2002 325 752 756 12364304 10.1136/bmj.325.7367.752 PMC128377

[B22] Pisters MF Veenhof C van Meeteren NLU Ostelo RW de Bakker DH Schellevis FG Dekker J Long-term effectiveness of exercise therapy in patients with osteoarthritis of the hip or knee: a systematic review Arthritis Rheum 2007 57 1245 1253 17907210 10.1002/art.23009

[B23] Marks R Allegrante JP Chronic osteoarthritis and adherence to exercise: a review of the literature J Aging Phys Act 2005 13 434 460 16301755 10.1123/japa.13.4.434

[B24] Roddy E Zhang W Doherty M Arden NK Barlow J Birrell F Carr A Chakravarty K Dickson J Hay E Hosie G Hurley M Jordan KM McCarthy C McMurdo M Mockett S O'Reilly S Peat G Pendleton A Richards S Evidence-based recommendations for the role of exercise in the management of osteoarthritis of the hip or knee – the MOVE consensus Rheumatol 2005 44 67 73 10.1093/rheumatology/keh399 15353613

[B25] Hillsdon M Foster C Thorogood M Interventions for promoting physical activity Cochrane Database Syst Rev 2005 1 CD003180 15674903 10.1002/14651858.CD003180.pub2 PMC4164373

[B26] Jordan JL Holden MA Mason EEJ Foster NE Interventions to improve adherence to exercise for chronic musculoskeletal pain in adults Cochrane Database Syst Rev 2010 1 CD005956 20091582 10.1002/14651858.CD005956.pub2 PMC6769154

[B27] Holden MA Nicholls E Young J Hay EM Foster NE UK-based physical therapists’ attitudes and beliefs regarding exercise and knee osteoarthritis: findings from a mixed methods study Arthritis Care Res 2009 61 1511 1521 10.1002/art.24829 19877105

[B28] Holden MA Nicholls EE Young J Hay EM Foster NE The role of exercise for knee pain: what do older adults in the community think? Arthritis Care Res 2012 64 1554 1564 10.1002/acr.21700 22511582

[B29] Holden MA Nicholls EE Young J Hay EM Foster NE Exercise and physical activity in older adults with knee pain: a mixed methods study Rheum 2014 in press 10.1093/rheumatology/keu333 PMC4334683 25187640

[B30] Bennell KL Hinman RS A review of the clinical evidence for exercise in osteoarthritis of the hip and knee J Sci Med Sport 2011 14 4 9 20851051 10.1016/j.jsams.2010.08.002

[B31] Marks R Knee osteoarthritis and exercise adherence: a review Curr Ageing Sci 2012 5 72 83 10.2174/1874609811205010072 21762086

[B32] WHO Adherence to long-term therapies: evidence for action 2003 Geneva, Switzerland: World Health Organisation Library

[B33] Fernandes L Hagen KB Bijlsma JW Andreassen O Christensen P Conaghan PG Doherty M Geenen R Hammond A Kjeken I Lohmander LS Lund H Mallen CD Nava T Oliver S Pavelka K Pitsillidou I da Silva JA de la Torre J Zanoli G Vliet Vlieland TP European League Against Rheumatism (EULAR) EULAR recommendations for the non-pharmacological core management of hip and knee osteoarthritis Ann Rheum Dis 2013 72 1125 1135 23595142 10.1136/annrheumdis-2012-202745

[B34] NICE. Osteoarthritis The care and management of adults with osteoarthritis 2008 National Institute of Health and Clinical Excellence

[B35] Rankin G Rushton A Olver P Moore A Chartered Society of Physiotherapy's identification of national research priorities for physiotherapy using a modified Delphi technique Physiotherapy 2012 98 260 272 22898585 10.1016/j.physio.2012.03.002

[B36] Von Korff M Ormel J Keefe FJ Dworkin SF Grading the severity of chronic pain Pain 1992 50 133 149 1408309 10.1016/0304-3959(92)90154-4

[B37] Thomas E Dunn KM Mallen C Peat G A prognostic approach to defining chronic pain: application to knee pain in older adults Pain 2008 139 389 397 18583051 10.1016/j.pain.2008.05.010

[B38] Dunn KM Croft PR Main CJ Von Korff M A prognostic approach to defining chronic pain: replication in a UK primary care low back pain population Pain 2008 135 48 54 17570585 10.1016/j.pain.2007.05.001

[B39] Jordan KP Kadam UT Hayward R Porcheret M Young C Croft P Annual consultation prevalence of regional musculoskeletal problems in primary care: an observational study BMC Musculoskelet Disord 2010 11 144 20598124 10.1186/1471-2474-11-144 PMC2903510

[B40] Bird SR Smith A James K Exercise benefits and prescriptions 1998 Devon: Stanley Thornes Ltd

[B41] McAuley E Jerome GJ Marquez DX Canaklisova S Blissmer B Exercise self-efficacy in older adults: social affective and behavioral influences Ann Behav Med 2003 25 1 7 12581930 10.1207/S15324796ABM2501_01

[B42] Bandura A Social foundations of thought and action: a social cognitive theory 1986 Englewood Cliffs, NJ: Prentice Hall

[B43] Deyo RA Compliance with therapeutic regimens in arthritis: issues, current status, and a future agenda Semin Arthritis Rheum 1982 12 233 244 6101215 10.1016/0049-0172(82)90063-4

[B44] Bravata DM Smith-Spangler C Sundaram V Gienger AL Lin N Lewis R Stave CD Olkin I Sirard JR Using pedometers to increase physical activity and improve health: a systematic review JAMA 2007 298 2296 2304 18029834 10.1001/jama.298.19.2296

[B45] Mutrie N Doolin O Fitzsimons CF Grant PM Granat M Grealy M Macdonald H MacMillan F McConnachie A Rowe DA Shaw R Skelton DA Increasing older adults' walking through primary care: results of a pilot randomized controlled trial Fam Pract 2012 29 633 642 22843637 10.1093/fampra/cms038 PMC3501246

[B46] Cameron LD Leventhal H The self-regulation of health and illness behaviour 2003 London: Routledge

[B47] Meichenbaum D Turk DC Facilitating treatment adherence: a practioner’s guidebook 1987 New York: Plenum Press

[B48] Bellamy N Buchanan WW Goldsmith CH Campbell J Stitt LW Validation study of WOMAC: a health status instrument for measuring clinically important patient relevant outcomes to antirheumatic drug therapy in patients with osteoarthritis of the hip or knee J Rheumatol 1988 15 1833 1840 3068365

[B49] O’Reilly SC Muir KR Doherty M Screening for pain in knee osteoarthritis: which question? Ann Rheum Dis 1996 55 931 933 9014590 10.1136/ard.55.12.931 PMC1010348

[B50] Trudeau J Van Inwegen R Eaton T Bhat G Paillard F Ng D Tan K Katz NP Assessment of pain and activity using an electronic pain diary and actigraphy device in a randomized, placebo-controlled crossover trial of Celecoxib in osteoarthritis of the knee Pain Pract 2014 10.1111/papr.12167 10.1111/papr.12167 24494935

[B51] Washburn RA Smith KW Jette AM Janney CA The physical activity scale for the elderly (PASE): development and evaluation J Clin Epi 1993 46 153 162 10.1016/0895-4356(93)90053-4 8437031

[B52] Borkovec TD Nau SD Credibility of analogue therapy rationales J Behav Ther Exp Psychiatry 1972 3 257 260

[B53] Devilly GJ Borkovec TD Psychometric properties of the credibility/expectancy questionnaire J Behav Ther Psychiatr 2000 31 73 86 10.1016/s0005-7916(00)00012-4 11132119

[B54] Broadbent E Petrie KJ Main J Weinmann J The brief illness perception questionnaire J Psychosom Res 2006 60 631 637 16731240 10.1016/j.jpsychores.2005.10.020

[B55] Resnick B Jenkins L Testing the reliability and validity of the self-efficacy for exercise scale Nurs Res 2000 49 154 159 10882320 10.1097/00006199-200005000-00007

[B56] Resnick B Reliability and validity of the outcome expectations for exercise scale-2 J Aging Phys Act 2005 13 382 394 16301750 10.1123/japa.13.4.382

[B57] Spitzer RL Kroenke K Williams JBW Lowe B A brief measure for assessing generalized anxiety disorder: The GAD-7 Arch Intern Med 2006 166 1092 1097 16717171 10.1001/archinte.166.10.1092

[B58] Kroenke K Spitzer RL The WJBW PHQ 9: validity of a brief depression severity measure J Gen Intern Med 2001 16 606 613 11556941 10.1046/j.1525-1497.2001.016009606.x PMC1495268

[B59] The EuroQoL Group EuroQoL-a new facility for the measurement of health-related quality of life Health Pol 1990 16 199 208 10.1016/0168-8510(90)90421-9 10109801

[B60] Department of Health Start Active, Stay Active. A report on physical activity for health from the four home countries’ Chief Medical Officers 2011 http://www.bhfactive.org.uk/userfiles/Documents/startactivestayactive.pdf

[B61] Cohen J Statistical power analysis for the behavioral sciences 1988 2 Hillsdale, New Jersey, Hove and London: Lawrence Erlbaum Associates

[B62] Terwee CB Roorda LD Dekker J Bierma-Zeinstra SM Peat G Jordan KP Croft P de Vet HC Mind the MIC: large variation among populations and methods J Clin Epidemiol 2010 63 524 534 19926446 10.1016/j.jclinepi.2009.08.010

[B63] Roberts C Roberts SA Design and analysis of clinical trials with clustering effects due to treatment Clin Trials 2005 2 152 162 16279137 10.1191/1740774505cn076oa

[B64] Boutron I Moher D Altman DG Schulz K Ravaud P for the CONSORT group Methods and processes of the CONSORT Group: example of an extension for trials assessing nonpharmacologic treatments Ann Intern Med 2008 148 W60 W67 18283201 10.7326/0003-4819-148-4-200802190-00008-w1

[B65] Boutron I Moher D Altman DG Schulz K Ravaud P for the CONSORT group Extending the CONSORT Statement to randomized trials of nonpharmacologic treatment: explanation and elaboration Ann Intern Med 2008 148 295 309 18283207 10.7326/0003-4819-148-4-200802190-00008

[B66] Craig P Dieppe P Macintyre S Michie S Nazareth I Petticrew M Developing and evaluating complex interventions: the new Medical Research Council guidance Int J Nurs Stud 2013 50 587 592 23159157 10.1016/j.ijnurstu.2012.09.010

[B67] Lewin S Glenton C Oxman A Use of qualitative methods alongside randomised controlled trials of complex healthcare interventions: methodological study BMJ 2009 339 b3496 19744976 10.1136/bmj.b3496 PMC2741564

[B68] O'Cathain A Thomas KJ Drabble SJ Rudolph A Hewison J What can qualitative research do for randomised controlled trials? A systematic mapping review BMJ Open 2013 3 e002889 10.1136/bmjopen-2013-002889 PMC3669723 23794542

[B69] Zwarenstein M Treweek S Gagnier JJ Altman DG Tunis S Haynes B Oxman AD Moher D CONSORT group, Pragmatic Trials in Healthcare (Practihc) group Improving the reporting of pragmatic trials: an extension of the CONSORT statement BMJ 2008 337 a2390 19001484 10.1136/bmj.a2390 PMC3266844

[B70] Moher D Hopewell S Schulz KF Montori V Gøtzsche PC Devereaux PJ Elbourne D Egger M Altman DG CONSORT CONSORT 2010 explanation and elaboration: updated guidelines for reporting parallel group randomised trials Int J Surg 2012 10 28 55 22036893 10.1016/j.ijsu.2011.10.001

[B71] Peduzzi P Henderson W Hartigan P Lavori P Analysis of randomized controlled trials Epidemiol Rev 2002 24 26 38 12119853 10.1093/epirev/24.1.26

[B72] Song J Semanik P Sharma L Chang R Hochberg M Mysiw W Bathon J Eaton C Jackson R Kwoh C Nevitt M Dunlop D Assessing physical activity in persons with knee osteoarthritis using accelerometers: data from the osteoarthritis initiative Arthritis Care Res 2010 629 1724 1732 10.1002/acr.20305 PMC2995807 20806273

[B73] Freedson PS Melanson E Sirard J Calibration of the computer science and applications, inc. accelerometer Med Sci Sports Exerc 1998 30 777 781 9588623 10.1097/00005768-199805000-00021

[B74] Joint Formulary Committee British National Formulary (online) London: BMJ Group and Pharmaceutical Press http://www.medicinescomplete.com

[B75] Curtis L Unit costs of health and social care 2013 PSSRU 2013 Canterbury: University of Kent

[B76] Department of Health NHS reference costs 2012/13 https://www.gov.uk/government/publications/nhs-reference-costs-2012-to-2013

[B77] Office of National Statistics Standard occupational classificaitons 2010 http://www.ons.gov.uk/ons/guide-method/classifications/current-standard-classifications/soc2010/index.html

[B78] Office for National Statistics Annual survey of hours and earnings (ASHE) 2013. Provisional results http://www.ons.gov.uk/ons/rel/ashe/annual-survey-of-hours-and-earnings/2013-provisional-results/index.html

[B79] Barber JA Thompson SG Analysis of cost data in randomised trials: an application of the non-parametric bootstrap Stat Med 2000 19 3219 3236 11113956 10.1002/1097-0258(20001215)19:23<3219::aid-sim623>3.0.co;2-p

[B80] Manca A Hawkins N Sculpher MJ Estimating mean QALYs in trial-based cost-effectiveness analysis: the importance of controlling for baseline utility Health Econ 2005 14 487 496 15497198 10.1002/hec.944

[B81] Fenwick E Claxton K Sculpher M Representing uncertainty: the role of cost-effectiveness acceptability curves Health Econ 2001 10 779 787 11747057 10.1002/hec.635

[B82] Plano Clark VL Schumacher K West CM Edrington J Dunn LB Harzstark A Melisko M Rabow MW Swift PS Miaskowski C Practices for embedding an interpretive qualitative approach within a randomized clinical trial J Mixed Meth Res 2013 10.1177/1558689812474372

[B83] Charmaz K Constructing grounded theory: A practical guide through qualitative analysis 2006 London: Sage

[B84] Saldana J Longitudinal qualitative research. Analysis change through time 2003 California: AltaMira Press

