# Supplementary material for: A multicentre, pragmatic, parallel group, randomised controlled trial to compare the clinical and cost-effectiveness of three physiotherapy-led exercise interventions for knee osteoarthritis in older adults: the BEEP trial protocol (ISRCTN: 93634563)
Source: BMC Musculoskelet Disord. 2014 Jul 27;15:254. doi: 10.1186/1471-2474-15-254 (PMC4123500; doi:10.1186/1471-2474-15-254)
Supplement: Additional file 1 — BEEP trial participant information leaflet. [file 1471-2474-15-254-S1.pdf]

KEELE UNIVERSITY

Arthritis Research UK
Providing answers today and tomorrow

NHS National Institute for Health Research

# The BEEP Study

BEEP

## Patient Information Leaflet

**<u>Version 2 20/12/10</u>**

REC NO: 10/H1017/45

We are inviting you to take part in a research study. This leaflet explains why the research is being done and what it will involve. Please take time to read the following information carefully, which will help you decide whether or not you wish to take part. Please ask the research team if there is anything that is not clear.

**What is the purpose of the study?**

Your General Practice and local Physiotherapy Department are working with a research team at the Arthritis Research UK Primary Care Centre at Keele University on a study that is trying to find out how best to treat people with knee problems.

Knee problems are common and can sometimes be disabling. Advice and exercise prescribed by a physiotherapist have been shown to be effective treatments for older adults with knee problems. However, there is a lack of evidence around the practical aspects of how exercise is delivered and maintained, including what is an appropriate amount of exercise and how to support individuals to continue to exercise in the longer-term.

The objective of the BEEP Study is to investigate how to improve the effectiveness of exercise for knee problems in older adults.

**Why have I been invited to participate in the study?**
You have been invited to take part in this study because you have a knee problem and may be suitable to participate in this study. In total 500 patients will be recruited from general practices and physiotherapy departments in the West Midlands and the North West area.

**Do I have to take part?**
Your involvement in the BEEP Study is purely **voluntary**. If you change your mind and decide to withdraw from the study at a later date your care will not be affected in any way. If you decide to take part you will be asked to sign and return a consent form and a questionnaire.

**What will happen to me if I take part?**
A study nurse will contact you, via the telephone. They will ask some general questions about your knee problem and your general health to check that you are eligible to take part in the study. If you are eligible and willing to take part, the study nurse will then talk to you about the study documents that have been sent to you. The study nurse will ask you if you have any questions or if there is anything you do not understand after reading this leaflet. They will explain the study to you in detail. Please do not hesitate to ask about anything that is not clear. We need to know that you fully understand what taking part in the study means to you. You will be asked to sign and return the written consent form and the baseline questionnaire to the research centre. Once the signed consent form and the completed baseline questionnaire are received you will be given an appointment with a member of your local physiotherapy team who will assess and treat your knee problem. Your GP will be informed of your participation in this study.

If you take part in the study you may also be asked to wear a physical activity monitor at certain times during the study, which is worn on a belt round your waist. You may also be asked to participate in a brief interview at the end of the study to explore your experiences of the treatment you received. However, you can decline to take part in either of these even if you would still like to take part in the study. Further information about these will be sent to you if you are asked to participate in these aspects of the study. We will also ask you to complete follow-up questionnaires at 3, 6, 9, 18 months and 3 years from the date you were allocated to your treatment, so that you can let us know how your knee problem is in the short and long term. If you agree to wear an activity monitor we will also ask you to wear it at each of these time points.

The BEEP study will compare 3 ways of supporting patients to exercise for their knee problem; a usual physiotherapy care package, an individually tailored lower-limb exercise programme, and a programme that focuses on helping patients to be more physically active in general. The details of these are provided on the next page.
We want to find out if helping people to find the right exercise routine and maintaining it over time results in improved function and reduced knee pain.

To find this out, you will be allocated to one of the following treatments:

**1. Usual physiotherapy care package**

This will involve the provision of a written information leaflet and advice on pacing, self-management and therapeutic exercise. This treatment package will consist of up to 4 consultations with a member of the physiotherapy team, each lasting approximately 20 to 30 minutes over a 12 week treatment period. These will be carried out at your local physiotherapy department.

**2. Individually tailored lower limb exercise programme**

This will involve starting and progressing an individually tailored exercise programme for your leg(s), which is supervised in the physiotherapy department and practiced at home. The exercise programme will be reviewed by a member of the physiotherapy team and changed at intervals according to principles of exercise for strengthening muscles, helping with movement and balance. You will also be given a written information leaflet and will be given the opportunity to discuss it. This treatment package will consist of up to 8 consultations each lasting approximately 20 to 30 minutes over a 12 week treatment period.

**3. General physical activity programme**

This will involve an exercise programme for your knee problem to begin with, and then a member of the physiotherapy team will help you to make changes to your general physical activity, over time. They will help you to identify ways to keep doing the exercises you have been recommended over the longer-term, and support you to integrate exercise and physical activities into your everyday activities. The exercise programme will be amended over time based on your experiences. You will also be given a written information leaflet and have the opportunity to discuss this with a member of the physiotherapy team. This treatment package will consist of up to 10 consultations each lasting approximately 20 to 30 minutes over a 6 month treatment period.

**What are the possible benefits of taking part?**

You may have already received some information and painkillers from your doctor. Exercise is one of the recommended ‘core’ treatments for older adults with knee problems and everyone who takes part in this study will receive exercise therapy, led by a member of the physiotherapy team. Although no direct benefit can be guaranteed for you, what we learn from the study should also help us to treat other people with knee problems in the future.

**What might be the risks?**
Serious risks are extremely unlikely. A member of the clinical team will check that there are no reasons why you should not receive any of the treatments. In people with knee problems exercise can sometimes cause temporary, mild soreness to the joints. The physiotherapist who is responsible for your treatment will be able to give you advice on how to manage these symptoms.

**Will my taking part in this study be kept confidential?**
All information that you supply will be dealt with in **strictest confidence**. We will inform your doctor of your involvement in the study, unless you indicate otherwise on the consent form. Each person who takes part in the BEEP study will be given a code number, so the data from the study will not have any identifiable names and addresses, and so cannot be traced back to you. This means that the data are anonymous, and on this basis may be used in other research studies. The questionnaires will be stored without identifiable names and addresses for twenty years in accordance with the Medical Research Council guidelines. The questionnaires will be stored in a secure place.

**What will happen to the results of the research study?**
After the study has finished and we have looked at the results we will publish the results in a medical journal. At this point we will send you a brief summary of the findings. We will also put up posters within your GP practice and physiotherapy department that provide brief summaries of the results. You will not be identified individually in any report or publication.

**Who is funding and organising the research?**
Keele University has been awarded funding from the National Institute for Health Research and the Arthritis Research UK to conduct this study.

**Who has reviewed the study?**
The North West 1 Research Ethics Committee – Cheshire, have reviewed this study (Ref:10/H1017/45). The study has also been reviewed by scientific experts, who assessed it before awarding funding.

**Contact for further information**
If you have any questions or would like further information about this project please contact the BEEP Study Co-ordinator, **Julie Young**, on **01782 733921**. If you have any questions or concerns about taking part in this research you can also contact the Patient Advice and Liaison Service (PALS) on (Tel: xxxxxxxxx**)**

Thank you for taking time to read this information leaflet.

N.B. The PALS number differed depending on the participating physiotherapy service
