# Supplementary material for: A multicentre, pragmatic, parallel group, randomised controlled trial to compare the clinical and cost-effectiveness of three physiotherapy-led exercise interventions for knee osteoarthritis in older adults: the BEEP trial protocol (ISRCTN: 93634563)
Source: BMC Musculoskelet Disord. 2014 Jul 27;15:254. doi: 10.1186/1471-2474-15-254 (PMC4123500; doi:10.1186/1471-2474-15-254)
Supplement: Additional file 2 — Copy of advice booklet provided to all BEEP participants. [file 1471-2474-15-254-S2.pdf]

The BEEP Study

Knee Problems

An Information Leaflet

Introduction

Knee problems are very common and can affect people in different ways. You have been given this information leaflet about knee problems because you are taking part in the BEEP study.

This leaflet is designed to help people with knee problems. To begin with, it will discuss what your knees do, and what may have caused the problems. It will then discuss how you can live with knee problems more easily.

At the back of this leaflet there are some addresses of organisations that provide some helpful advice for people who have knee problems.

What do knees do and how do they work?

The knee joint is the largest and most complex joint in the body. Your knees provide stable support for your body and allow your legs to bend and straighten. Both flexibility and stability are needed for standing and for activities such as walking, crouching and jumping. Your knees are also really important in weight-bearing.

In order to keep the joint stable, your knee consists of many supporting and moving parts, which include: bones, cartilage, muscle, ligaments,

and tendons. Any one of these parts can be involved in knee problems.

Here is a simple diagram of the knee joint showing the main bones, muscles and tendons.

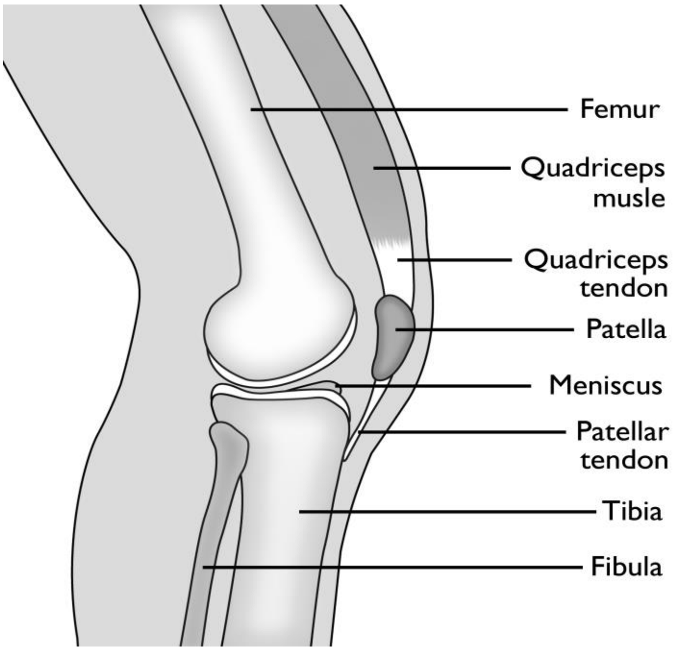

What can cause a knee problem?

Many things can cause and increase the risk of knee problems. The risk of having a knee problem increases as we get older and is more common in females and people that are overweight.

There are a few main types of knee problems:

- **Osteoarthritis**
  This is the most common type of knee joint disorder. It tends to develop over time and is the result of thinning or loss of cartilage in the joint,

that then affects the whole joint, including muscles, tendons and ligaments. Cartilage is the tissue that is found at the ends of the bones that helps to ensure that the bones move smoothly against each other and acts as a shock-absorber. Osteoarthritis tends to progress slowly and the condition can remain stable for many years without getting worse. It is a clinical diagnosis based on the symptoms and signs you present with.

- **Mechanical**

  Some knee problems result from injury, such as a direct blow or sudden movements that put added strain on your knees, and thus resulting the knee to strain beyond its normal range of movements. Previous knee injuries can lead to osteoarthritis.

- **Inflammatory Knee Problems**

  Inflammation that occurs in certain rheumatic diseases (e.g. rheumatoid arthritis) can damage the knee.

What are the main issues with having a knee problem?

Some people experience problems with one knee while others experience problems with both. Knee problems can cause swelling, pain, stiffness and

make you feel tired or irritable, which may influence your ability to get around and carry out daily tasks.

Although knee problems are very common, it affects different people in different ways.

The most common features include:

- Knee pain that tends to gradually develop, often described as a deep, aching pain. The pain is usually worse after periods of activity and some people find they have pain even when they are at rest.
- Knee stiffness after a period of inactivity, for example first thing in the morning. This usually passes within 30 minutes.
- Muscle weakness in the large muscles of the leg. Due to reduced use, this weakness can progress over time and the muscles may visibly ‘waste’.

Sometimes, people also experience swelling in and around the knee, cracking or grating noises as the knee moves, general tenderness around the knee and changes in the alignment of the bones around the knee.

How to live with your knee problem more easily

There are some reasons for knee problems that we cannot alter, for example, getting older and our family history/genetic make-up. However, there are a number of things that you can influence and these will help your knee problem:

- **Ease pain and discomfort**
- **Reduce the stiffness**
- **Decrease the chance of the problem worsening**

This can be achieved through:

- **Exercise**
  Joints and muscles **have to be moved** to keep them healthy, even if you have pain; too much rest may be **bad** for your joints. The benefits of exercise can include gaining more control over your knee problem and improving your ability to do your daily activities. Exercise will also help with losing weight and feeling better about yourself.

  Exercises that can be done at home, requiring little or no equipment will help with pain and everyday activities. Your physiotherapist will provide you with advice about exercise and suggest which exercises you should try. The exercises may be specifically for your knee or

may be focused on increasing your general physical activity levels.

Physiotherapists are experts at teaching exercises, which may strengthen your knees, increase the movement in your knees and enable you to do your daily activities. Physiotherapists can help to set realistic goals and advise you how to balance your daily activities so that you can manage better.

Regular, moderate exercise has many benefits. As well as improvement to the knee joint and the surrounding muscles, it promotes overall health and fitness, which gives you more energy, helps with weight control, helps you sleep better, reduces stress and improves your mood.

Exercise needs to be gradually changed over time in order to strengthen the muscles and ensure you are getting the most benefit. Some mild discomfort during and after exercise is to be expected, this usually means you are working the area. Remember to start slowly and make it fun!

- **Losing weight**
  If you are overweight, although you may find it difficult, losing weight will help with your knee problem. Studies have shown that people who

have lost excess weight have had less knee problems than those who have not. Try to eat a healthy diet by increasing the amount of fruit and vegetables and cutting down on food that has a high fat and sugar content.

- **Wearing the right shoes**
  Flat shoes or ones with a low heel and shoes that are cushioned and broad with support for arches of the foot should help with your knee problem. Many good training or running shoes have these features.

- **Pacing yourself**
  Pain can change from day to day and it is normal to try to do as much as you can on a ‘good’ day. It is important to pace your activities, by breaking them down into manageable chunks and swapping between different types of activity, or between activity and rest. The best advice for people with knee problems is ‘little and often’: a little rest, followed by a little activity. For example, you can alternate short periods of activity, such as exercise, housework or gardening, with short periods of rest.

  It helps to prioritise your activities and break them up into manageable amounts spread throughout the day, keeping to this routine even when your knee is okay.

- **Using heat and cold**
  You may find that heat helps with the pain and stiffness caused from your knee problem. Use a hot water bottle wrapped in a towel, or a heating pad, for 15 minutes at a time, ensuring that you leave at least 2 hours between applications.

  If heat does not help then try cold ice packs, e.g. a bag of frozen peas wrapped in a damp cloth, applied for up to 20 minutes at a time. Make sure you leave at least 2 hours between applications.

  There are also many hot and cold sprays and creams available at the chemist, which can be applied to your knee.

  Remember that heat and ice can burn your skin so check the skin regularly.

- **Walking aids**
  You may find that using a walking stick or cane helps reduce some of the weight going through your knee and thus helps with the pain. Your physiotherapist can advise you about what is best and help you to use a walking aid in the most effective way.

- **Pain relievers**
  There are a number of pain relievers that can help you deal with your symptoms. These include simple over the counter medications and those prescribed by a health care professional. There are two main areas where drugs can be used to help your knee problem: to decrease pain and reduce inflammation.

  Paracetamol is a very safe and effective painkiller. Two tablets (total of 1gram) can be taken safely up to four times daily and may give relief that lasts up to 6 hours. This may be all you need to control your pain caused by your knee problem.

  Anti-inflammatory tablets, such as ibuprofen, can reduce pain and inflammation, but can also cause side effects such as stomach upsets. Anti-inflammatory drugs are also available as creams or gels to rub into the skin overlying the joint. These can also help pain and inflammation and do not cause as many side effects.

  Other medications can be prescribed by your GP. It is important to talk to your doctor if you feel that the medicines you are currently taking are not controlling your pain.

What else can I do?

- When sitting for long periods remember to move your legs.
- **Be Positive** about your knee problem and don’t be afraid to help yourself.
- Remember just because something doesn’t help someone else it doesn’t mean it will not help you.

Questions you want to ask

Please make a note of any questions you may want to ask your physiotherapist in the space below and they will be happy to answer them.

Useful Information

You may wish to note any useful information given by your physiotherapist in the space below and use it at a later date.

Some Useful Addressees

Arthritis Research UK: a charity promoting research into the cause, treatment and cure of arthritic conditions
PO Box 177
Chesterfield
Derbyshire
S41 7TQ
Tel: 0870 850 5000
www.arthritisresearchuk.org

Arthritis Care: an organisation that provides information and support.
18 Stephenson Way
London
NW1 2HD
Tel: 020 7380 6500
Helpline: 0808 800 4050
www.arthritiscare.org.uk
