# Supplementary material for: A multicentre, pragmatic, parallel group, randomised controlled trial to compare the clinical and cost-effectiveness of three physiotherapy-led exercise interventions for knee osteoarthritis in older adults: the BEEP trial protocol (ISRCTN: 93634563)
Source: BMC Musculoskelet Disord. 2014 Jul 27;15:254. doi: 10.1186/1471-2474-15-254 (PMC4123500; doi:10.1186/1471-2474-15-254)
Supplement: Additional file 3 — Copy of lower limb exercise diary. [file 1471-2474-15-254-S3.pdf]

My Knee Exercise Diary

Name:

Study ID:

Physiotherapist:

Date:

This diary is for you to keep a record of the knee exercises that you do, as advised by your physiotherapist.

It is to help you and your physiotherapist see if you are managing to complete your exercises, so it works best if you are honest.

There are no right and wrong answers.

Instructions

Please:

1. Try to fill your diary in regularly.

2. Every time you complete a set of knee exercises place a tick in the appropriate box.

3. Record how hard you felt the exercises were (using the scale on the next page).

4. There is a comments box for you to write any thoughts or feelings you have each day. For example, you could record if the exercises are becoming easier, or if you find any exercises particularly difficult.

If you did not manage to complete any exercises that day you can note why, and your physiotherapist can help you to work around any obstacles you uncover.

Below is an example of what part of a week might look like:

	**Sets of each exercise completed (please tick)**						**How the exercises feel**	**Comments**
	**Exs. 1**	**Exs. 2**	**Exs. 3**	**Exs. 4**	**Exs. 5**	**Exs. 6**		
**Monday**	✓ ✓	✓	✓ ✓	✓ ✓	✓ ✓	✓ ✓	*5*	*Exercise 1 was easy, 2 was difficult*

‘How the exercises feel’ scale

1. Very, very easy /
   no problem
2. Very easy
3. Fairly easy
4. **Moderate/
   beginning to feel hard**
5. **Fairly hard**
6. **Hard**
7. Very hard
8. Very, very hard
9. Extremely hard
10. Maximum

The exercises should feel between level 4 (“moderate/ beginning to feel hard”) and level 6 (“Hard”). Below this you are not getting the maximum benefit and above this you are working harder than you need to.

REMEMBER

- It is very important to build up your exercises gradually
- Work at a level/pace that is right for you
- After exercise, it is normal to experience some discomfort around your knee and in your leg muscles, and this may last for a couple of days. BUT, if the discomfort is severe, or lasts for longer than this, reduce your exercises and contact your physiotherapist.

Week commencing:……/……./……./

	Sets of each exercise completed (please tick)						How the exercises feel (use scale, p 3)	Comments
	Exs. 1	Exs. 2	Exs. 3	Exs. 4	Exs. 5	Exs. 6		
**Monday**								
**Tuesday**								
**Wednesday**								
**Thursday**								
**Friday**								
**Saturday**								
**Sunday**								

Week commencing:……/……./……./

	Sets of each exercise completed (please tick)						How the exercises feel (use scale, p 3)	Comments
	Exs. 1	Exs. 2	Exs. 3	Exs. 4	Exs. 5	Exs. 6		
**Monday**								
**Tuesday**								
**Wednesday**								
**Thursday**								
**Friday**								
**Saturday**								
**Sunday**								

Week commencing:……/……./……./

	Sets of each exercise completed (please tick)						How the exercises feel (use scale, p 3)	Comments
	Exs. 1	Exs. 2	Exs. 3	Exs. 4	Exs. 5	Exs. 6		
**Monday**								
**Tuesday**								
**Wednesday**								
**Thursday**								
**Friday**								
**Saturday**								
**Sunday**								

Week commencing:……/……./……./

	Sets of each exercise completed (please tick)						How the exercises feel (use scale, p 3)	Comments
	Exs. 1	Exs. 2	Exs. 3	Exs. 4	Exs. 5	Exs. 6		
**Monday**								
**Tuesday**								
**Wednesday**								
**Thursday**								
**Friday**								
**Saturday**								
**Sunday**								
