# Supplementary material for: A multicentre, pragmatic, parallel group, randomised controlled trial to compare the clinical and cost-effectiveness of three physiotherapy-led exercise interventions for knee osteoarthritis in older adults: the BEEP trial protocol (ISRCTN: 93634563)
Source: BMC Musculoskelet Disord. 2014 Jul 27;15:254. doi: 10.1186/1471-2474-15-254 (PMC4123500; doi:10.1186/1471-2474-15-254)
Supplement: Additional file 4 — Summary of contents of adherence enhancing tool kit. [file 1471-2474-15-254-S4.doc]

# Webappendix 4: Summary of contents of adherence enhancing toolkit

# Section 1 – Information for physiotherapists

- Instructions for using the adherence enhancing toolkit
- Background information about exercise, knee pain in older adults and adherence
- CD containing:
  - electronic version of the Toolkit

# Section 2 – Educational aids

#

- The BEEP advice and information leaflet
- TENS guide
- Medication guide
- Walking guide
- Intensities for common activities
- Exercise and chronic conditions
- Useful website addresses for patient information
- Examples of other information leaflets
- Frequently asked questions
- Instructions for PhysioTools

# Section 3 – Behavioural aids

- Pedometer instructions and pedometers
- Physiotools software
- Visual feedback chart
- Reminder postcard
- Graded activity sheet
- Physical activity diary
- Knee exercise diary
- How to measure heart rate guide

**Section 4 – Cognitive behavioural aids**

- Questions to elicit health related beliefs
- Identifying barriers/ facilitators to exercise
- SMART goal setting
- Exercise and physical activity contracts
- Rulers (readiness ruler, confidence ruler, importance ruler)
- Set-back plan sheet

# Section 5 – Local lifestyle change opportunities

- Exercise and physical activity opportunities in the local area (developed for local areas by participating physiotherapists)
