# Supplementary material for: A multicentre, pragmatic, parallel group, randomised controlled trial to compare the clinical and cost-effectiveness of three physiotherapy-led exercise interventions for knee osteoarthritis in older adults: the BEEP trial protocol (ISRCTN: 93634563)
Source: BMC Musculoskelet Disord. 2014 Jul 27;15:254. doi: 10.1186/1471-2474-15-254 (PMC4123500; doi:10.1186/1471-2474-15-254)
Supplement: Additional file 5 — Copy of physical activity diary. [file 1471-2474-15-254-S5.pdf]

My physical activity diary

Name:

Study ID:

Physiotherapist:

Date:

This diary is for you to keep a record of the physical activity that you do.

It is to help you and your physiotherapist see if you are managing to increase your physical activity level, so it works best if you are honest.

There are no right and wrong answers.

Instructions

Please:

1. Try to fill your diary in regularly.

2. Record the type of activity that you completed (e.g. walking or swimming), how long you spent doing the activity (in minutes), and how hard the activity was (use the scale on the next page).

3. **If your physiotherapist has given you a step monitor,** record how many steps you have done in a day.

4. There is a comments box for you to write any thoughts or feelings you have. You could record if anything went particularly well, or if you found anything difficult. If you did not manage to complete any activity that day you can note why, and your physiotherapist can help you to work around any obstacles you uncover.

Below is an example of what part of a week might look like:

**Day**	**Activities**	**Time (min)**	**How the activity feels**	**Daily step count**	**Comments**
**Monday**	*Walked to shops*<br><br>*Swimming*	*15*<br><br>*20*	*4*<br><br>*5*	*2100*	 <br>*I enjoyed swimming*

‘How the physical activity feels’ scale

1. Very, very easy / no problem
2. Very easy
3. Fairly easy
4. **Moderate/ beginning to feel hard**
5. **Fairly hard**
6. **Hard**
7. Very hard
8. Very, very hard
9. Extremely hard
10. Maximum

The physical activity should feel between level 4 (“moderate/ beginning to feel hard”) and level 6 (“Hard”). Below this you are not getting the maximum benefit and above this you are working harder than you need to.

REMEMBER

- It is very important to build up your activity gradually
- Work at a level/ pace that is right for you
- Do not try and compete with other people
- When you increase your activity it is normal to experience some pain around your knee and in your leg muscles, and this may last for a couple of days. BUT, if the pain is severe, or lasts for longer than this, reduce your activity and contact your physiotherapist.
- STOP immediately and go and see you doctor if you:
  - Feel sick/ dizzy
  - Experience any chest pain
  - Experience any palpitations

Week commencing: ……/……./……./

Day	Activities	Time (min)	How the activity feels (use scale p 3)	Daily step count	Comments
**Monday**					
**Tuesday**					
**Wednesday**					
**Thursday**					
**Friday**					
**Saturday**					
**Sunday**					

Week commencing: ……/……./……./

Day	Activities	Time (min)	How the activity feels (use scale p 3)	Daily step count	Comments
**Monday**					
**Tuesday**					
**Wednesday**					
**Thursday**					
**Friday**					
**Saturday**					
**Sunday**					

Week commencing: ……/……./……./

Day	Activities	Time (min)	How the activity feels (use scale p 3)	Daily step count	Comments
**Monday**					
**Tuesday**					
**Wednesday**					
**Thursday**					
**Friday**					
**Saturday**					
**Sunday**					

Week commencing: ……/……./……./

Day	Activities	Time (min)	How the activity feels (use scale p 3)	Daily step count	Comments
**Monday**					
**Tuesday**					
**Wednesday**					
**Thursday**					
**Friday**					
**Saturday**					
**Sunday**					
